# Landscape attributes governing local transmission of an endemic zoonosis: Rabies virus in domestic dogs

**DOI:** 10.1111/mec.14470

**Published:** 2018-01-29

**Authors:** Kirstyn Brunker, Philippe Lemey, Denise A. Marston, Anthony R. Fooks, Ahmed Lugelo, Chanasa Ngeleja, Katie Hampson, Roman Biek

**Affiliations:** ^1^ Institute of Biodiversity, Animal Health and Comparative Medicine University of Glasgow Glasgow UK; ^2^ The Boyd Orr Centre for Population and Ecosystem Health University of Glasgow Glasgow UK; ^3^ Animal and Plant Health Agency Addlestone UK; ^4^ Department of Microbiology and Immunology KU Leuven – University of Leuven Leuven Belgium; ^5^ Department of Veterinary Medicine and Public Health Sokoine University of Agriculture Morogoro United Republic of Tanzania; ^6^ Tanzania Veterinary Laboratory Agency Dar es Salaam United Republic of Tanzania

**Keywords:** domestic dog, endemic zoonotic disease, landscape heterogeneity, phylogeography, rabies, spatial diffusion

## Abstract

Landscape heterogeneity plays an important role in disease spread and persistence, but quantifying landscape influences and their scale dependence is challenging. Studies have focused on how environmental features or global transport networks influence pathogen invasion and spread, but their influence on local transmission dynamics that underpin the persistence of endemic diseases remains unexplored. Bayesian phylogeographic frameworks that incorporate spatial heterogeneities are promising tools for analysing linked epidemiological, environmental and genetic data. Here, we extend these methodological approaches to decipher the relative contribution and scale‐dependent effects of landscape influences on the transmission of endemic rabies virus in Serengeti district, Tanzania (area ~4,900 km^2^). Utilizing detailed epidemiological data and 152 complete viral genomes collected between 2004 and 2013, we show that the localized presence of dogs but not their density is the most important determinant of diffusion, implying that culling will be ineffective for rabies control. Rivers and roads acted as barriers and facilitators to viral spread, respectively, and vaccination impeded diffusion despite variable annual coverage. Notably, we found that landscape effects were scale‐dependent: rivers were barriers and roads facilitators on larger scales, whereas the distribution of dogs was important for rabies dispersal across multiple scales. This nuanced understanding of the spatial processes that underpin rabies transmission can be exploited for targeted control at the scale where it will have the greatest impact. Moreover, this research demonstrates how current phylogeographic frameworks can be adapted to improve our understanding of endemic disease dynamics at different spatial scales.

## 1 INTRODUCTION

The landscape occupied by a pathogen is spatially complex (McCallum, 2008; Ostfeld, Glass, & Keesing, 2005; Real & Biek, 2007), and spatial heterogeneities influence pathogen spread (Grenfell, Bjørnstad, & Kappey, 2001; Keeling et al., 2001; Meentemeyer, Haas, & Václavík, 2012; Meentemeyer et al., 2011; Pavlovsky & Levine, 1966; Real & Biek, 2007). Topographical features like rivers and mountain ranges and socio‐ecological characteristics like road networks can impede or facilitate host movement, and influence host distributions and densities. Meanwhile, the implementation of control measures such as vaccination affects the susceptibility of host populations. The interaction between natural and anthropogenic landscapes is an important aspect of infection dynamics for pathogens of both humans and animals (Bourhy et al., 2016; Gire et al., 2014; Lemey et al., 2014; Pybus, Tatem, & Lemey, 2015; Talbi et al., 2010). Understanding the scale over which landscape attributes act on transmission mechanisms and how they, individually and in combination, influence the spread of infection is a major challenge (Levin, 1992; Viboud et al., 2006; Wu, 2004).

Direct transmission of infection is rarely observed, but pathogen genetic data provide information from which drivers of transmission can be inferred. Viral phylogeographic analysis exploits genetic information to explore how interactions between evolutionary and spatial processes give rise to contemporaneous viral geographical distributions. Its application has uncovered important aspects of infectious disease spread including the global migration dynamics underlying human influenza H3N2 transmission (Bedford, Cobey, Beerli, & Pascual, 2010; Lemey et al., 2014), the impact of border closures during the 2013–2016 West African Ebola outbreak (Dudas et al., 2017) and variation in epidemic raccoon rabies spread through space and time (Lemey, Rambaut, Welch, & Suchard, 2010a,b). Notably, most phylogeographic studies focus on epidemic spread. In contrast, endemic pathogens have received less attention despite evidence of persisting phylogeographic structure and discernible patterns of dispersal (Bourhy et al., 2016; Brunker et al., 2015; Raghwani et al., 2011). Increasingly, combined genetic and epidemiological/environmental data are being used to resolve our understanding of complex pathogen dynamics (Bedford et al., 2010; Faria et al., 2014; Lemey et al., 2014; Trovão et al., 2015). Analytical tools to incorporate spatial heterogeneity and exploit landscape genetic approaches are rapidly evolving as demand grows for methods to analyse spatially resolved and linked epidemiological, environmental and genetic data sets (Brockmann & Helbing, 2013; Dellicour, Rose, & Pybus, 2016; Lemey et al., 2014). Such an integrated approach, drawing on these data, could elucidate the contribution of different processes underlying endemic pathogen transmission dynamics and their scale dependence (Baele, Suchard, Rambaut, & Lemey, 2016).

Dog‐mediated rabies is a substantial but neglected public health priority, responsible for around 59,000 human deaths globally every year (Hampson et al., 2015). The causative agent, rabies virus (RABV), is a rapidly evolving negative‐sense RNA virus that causes a fatal neurological infection in mammalian hosts. Domestic dogs are responsible for over 99% of all human deaths from rabies, which occur predominantly in Asia and Africa (WHO, 2013). Although mass dog vaccination has repeatedly been shown to effectively control rabies in domestic dog populations (Cleaveland, Kaare, Knobel, & Laurenson, 2006; Cleaveland, Kaare, Tiringa, Mlengeya, & Barrat, 2003; Hampson et al., 2007; Morters et al., 2013; Townsend et al., 2013), lingering doubts about the role of wildlife in maintaining infection, and the perceived need to reduce dog populations, affect the implementation of control measures (Lembo et al., 2010). Improved understanding of the local drivers of RABV spread in domestic dog populations could therefore support rabies control efforts, especially as they focus towards the goal of elimination (Hampson et al., 2016; Lankester et al., 2014; Mpolya et al., 2017).

As a directly transmitted pathogen, RABV is inevitably shaped by landscape influences on the movement, distribution, density and susceptibility of hosts (Table 1). Well‐studied rabies epidemics in wildlife populations exhibit irregular waves of spread driven by key landscape features and human‐mediated long‐distance translocations (Russell, Real, & Smith, 2006; Smith, Lucey, Waller, Childs, & Real, 2002). Previous studies indicate that phylogeographic structure of dog‐mediated rabies is similarly shaped by an interplay of physical and human geography (Bourhy et al., 1999, 2008; Brunker, Hampson, Horton, & Biek, 2012; Brunker et al., 2015; Talbi et al., 2009, 2010). For example, physical barriers delineate major canine RABV clades (Bourhy et al., 2008), while road and trade networks facilitate human‐mediated dispersal (Brunker et al., 2015; De Mattos et al., 1999; Denduangboripant et al., 2005; Talbi et al., 2010; Tenzin, Dhand, Dorjee, & Ward, 2011). But, much less is known about landscape drivers on the local spread and persistence of endemic dog rabies.

**Table 1:** Details of the landscape attributes hypothesized to influence rabies virus spread in the Serengeti district, Tanzania. Village areas ranged from 9 to 220 km^2^, and all landscape attributes were scaled to a 100 m resolution (100 × 100 m grid cells). Resistance values were assigned to each grid cell to represent the presumed effect of each attribute on rabies virus diffusion, that is, as a facilitator or barrier to spread. A barrier effect is represented by high values denoting greater resistance to movement, whereas facilitators are assigned small resistance values denoting greater ease of movement (calculated as the reciprocal of a presumed conductance value, e.g., a conductance of 100 is represented by a resistance value of 0.01)

Mechanism	Attribute	Hypothesized effect on dispersal	Rationale	Measurement	Range of resistance values	Data Source
Host demography	Dog density	Facilitator	Density‐dependent transmission often assumed for directly transmitted pathogens such as RABV (Cross et al., 2013; Ferrari, Perkins, Pomeroy, & Bjørnstad, 2011; Morters et al., 2013).	Isotropic Gaussian smoothing kernel applied to census dog counts in grid cells.	0.034–10	Human and dog population census (Sambo et al., 2017)
	Dog presence	Facilitator	Dog population distribution and possible movement routes (Beyer et al., 2011; Bourhy et al., 2016). Areas without dogs (or humans) are expected to be occupied by wildlife, which are considered to be nonmaintenance (i.e., dead end) hosts in this system (Lembo et al., 2007, 2008).	Dog presence/absence per cell.	0.1–1	Human and dog population census (Sambo et al., 2017)
	Elevation	Barrier	Typically lower human (and dog) densities at higher elevations (Cohen & Small, 1998).	90 m resolution resampled to 100 m resolution	1,164–1,741	Digital elevation model (DEM) from NASA Shuttle Radar Topology Mission data http://srtm.usgs.gov/index.php
Host movement	Human: dog ratio (HDR)	Barrier	Measure of human intervention: in areas with higher HDR, rabid dogs may be more rapidly caught/killed.	Village‐level HDRs from human and dog counts.	3.39–12	Human and dog population census (Sambo et al., 2017)
	Rivers	Barrier	Barriers to dog movement unless movement is facilitated by human activity (60,61).	Shape file rasterized	1–1,000	http://www.glcn.org/activities/africover_en.jsp
	Roads	Facilitator	Presence of humans (and dogs) close to roads/dog behaviour influenced by roads (e.g., food, movement)/human‐mediated transport.	Shape file rasterized	0.001–1	http://www.glcn.org/activities/africover_en.jsp
	Slope	Barrier	Steepness acts as a physical impediment to host movement.	90 m resolution DEM resampled to 100 m resolution	1–1.24	Estimated from resampled DEM (see above)
	Uniform landscape	Barrier	Dog movements expected to follow an isolation‐by‐distance pattern (Wright, 1943), that is, a null model for comparison.	Uniform grid	1	NA
Host susceptibility	Average vaccination coverage	Barrier	Vaccination coverage increases herd immunity, reducing transmission and disease incidence	Annual vaccination coverage from 2004 to 2013 averaged and aggregated at village level	6.43–100	This study
	Campaigns over 10‐year period	Barrier	High coverage, repeat campaigns are most effective for reducing transmission and for disease elimination (Ferguson et al., 2015; Townsend et al., 2013).	Number of vaccination campaigns with at least 10% coverage per village from 2004 to 2013	2–14	This study
	Susceptible host density	Facilitator	Resistance surface incorporating vaccination of the dog population.	Same as total density (see above)	0.037–10	This study

As control measures such as vaccination and population reduction contribute to landscape heterogeneity, this framework also provides a means to determine both the most appropriate form of control and the impact of control measures. Culling continues to be used as a response to rabies outbreaks in many parts of the world (Putra, Hampson, & Girardi, 2013; Windiyaningsih, Wilde, Meslin, Suroso, & Widarso, 2004). Although transmission of pathogens such as rabies is often considered to depend on population density (Anderson & May, 1991), empirical evidence suggests that dog density has little effect on RABV transmission (Hampson et al., 2009; Morters et al., 2013). Moreover, dog population reduction alone has proven ineffective for rabies control (Lee et al., 2001; WHO, 2013; Windiyaningsih et al., 2004). Phylogeographic signatures may elucidate the relative roles of dog population structure and density on RABV, and of vaccination. These insights are critical to determining what interventions will be most effective at the scale of their implementation.

Integrating genetic, environmental and population data within phylogeographic frameworks offers the opportunity to quantify how, individually and in combination, different landscape attributes influence the local transmission processes that underpin endemic circulation of dog‐mediated rabies. Here, integrated and flexible phylogeographic frameworks are used to decipher the relative contribution and scale‐dependent effects of landscape influences on transmission dynamics of endemic RABV in Serengeti district, Tanzania (area ~4,900 km^2^). Based on a unique data set of genetic, epidemiological and landscape data, including vaccination coverage and dog density, we aim to elucidate the key mechanisms underlying the local spread of RABV.

## 2 MATERIALS AND METHODS

### 2.1 Sequence data

In total, 152 whole‐genome sequences were used for this analysis, including 119 new sequences. Of these 119, 27 partial sequences previously submitted to GenBank were updated to whole‐genome sequences under the same accession number (Brunker et al., 2015). The remaining 33 whole‐genome sequences from the previous study were also used. Sample details, including epidemiological data, sequence details and GenBank accession numbers, are listed in Table S3.

Brain samples were obtained from rabid animals in the Serengeti district of northwest Tanzania from 2004 to 2013, along with the GPS location, and date symptoms were observed for each case. Samples were processed at the Animal & Plant Health Agency in Weybridge (APHA) as described in Brunker et al. (2015) (except for five samples sequenced by 454 pyrosequencing, see Methods S1 and Table S3). In brief, total RNA was extracted from brain material using TRIzol and subject to two depletion stages to reduce the proportion of host genetic material. Host genomic DNA was depleted using the on‐column DNase treatment in a RNeasy plus mini kit (Qiagen) followed by ribosomal RNA depletion in a reaction with Terminator 5′‐phosphate‐dependent exonuclease (Epicentre), which selectively digests RNA with a 5′‐monophosphate end. Depleted RNA was subjected to a round of purification using the RNeasy plus mini kit without DNase treatment and eluted in 30 μl molecular‐grade water. Double‐stranded cDNA was transcribed using a cDNA synthesis system kit with random hexamers (Roche) and libraries for sequencing prepared via a Nextera‐XT protocol (Illumina). Libraries were sequenced on an Illumina MiSeq (Medical Research Council Centre for Virus Research at the University of Glasgow, UK) or NextSeq platform (Glasgow Polyomics centre at the University of Glasgow, UK) with 150‐bp paired‐end reads. Raw reads were processed as described in Brunker et al. (2015), and SNPs were filtered in GATK according to strand bias (FS>60, SOR>4), mapping quality (MQ<40, MQRankSum< (‐)12.5), read position (ReadPosRankSum<(‐)8.0) and depth of coverage (DP<5). Filtered SNPs were called with a 75% consensus rule (ambiguous bases were given an IUPAC code), and genome positions with no coverage or covered by less than two reads were labelled “N.”

### 2.2 Landscape

The study landscape was defined as a spatial grid encompassing the Serengeti district (spatial extent: *x*_min_ = 637,638.2, *y*_min_ = 9,757,825.5, *x*_max_ = 705,238, *y*_max_ = 9,835,425) with a resolution of 100 m. Landscape attributes (note: the term “attribute” is used as a general descriptor for any landscape feature or process that may affect viral dispersal) were characterized as resistance surfaces with grid cells assigned resistance values according to the assumed facilitating or impeding impact of an attribute on RABV diffusion. Data sources used for each landscape attribute are detailed in Table 1. Specifically, a complete census of the human and dog population in the Serengeti district, involving collecting GPS locations for each household and the vaccination status of each dog, was conducted over a 7‐year period, as described in Sambo et al. (2017). This census was used to populate resistance matrices for dog presence, dog density and susceptible dog density.

Resistance landscapes for each attribute were constructed individually, with resistance values justified according to evidence from previous studies (Table 1). For example, rivers have previously been identified as barriers to RABV dispersal, and cells containing a river were therefore assigned a high resistance value. Landscape attributes assumed to facilitate diffusion were given resistance values according to the reciprocal of their assumed conductance; for example, roads were assigned an arbitrary conductance of 1,000 giving a resistance value of 0.001. Cells with no attribute were assigned a resistance value of one to represent a uniform landscape. A null model of isolation by distance (IBD) was created, where all cell values were set to one.

Circuitscape (Shah & McRae, 2008) was used to generate a matrix of pairwise resistance distances between all rabies sample locations for each landscape‐informed resistance surface. The program uses a combination of circuit and graph theory to model connectivity according to the effective resistance between pairs of points or focal regions (see McRae, Dickson, Keitt, and Shah (2008) for a detailed review). Landscape grids are converted to graphs where each cell is represented by a node and connections by undirected weighted edges. Resistance (i.e., edge weights) between two nodes was calculated as the average per‐cell resistance value. An advantage to circuit theory methodology is that multiple connections between nodes can be considered (in this analysis, eight neighbours were considered for each node) accounting for the effect of multiple pathways connecting points and producing an effective resistance distance (McRae et al., 2008).

**Figure 1:**
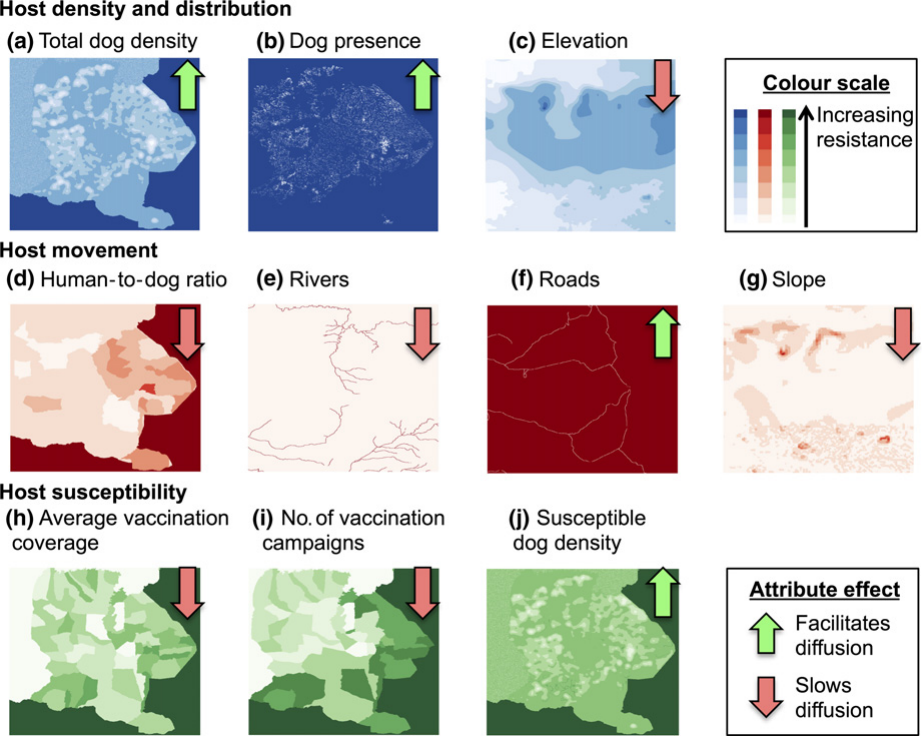
Resistance surfaces for landscape attributes hypothesized to influence rabies virus movement in the Serengeti district. Host density and distribution (a–c), host movement (d–g) and host susceptibility influenced by vaccination (h–j). Block arrows indicate whether the attribute was considered a facilitator (green) or barrier (red) to viral movement

Details of the different landscape attributes tested are shown in Table 1, and final resistance landscapes are shown in Figure 1. Details on the construction of resistance surfaces for each attribute can be found in the Methods S1.

### 2.3 Empirical tree distribution

To overcome the computationally intensive task of exploring phylogenetic tree space repeatedly, in each set of analyses, a posterior distribution of timescaled trees was inferred from sequence data once using beast v1.8.1 (Drummond & Rambaut, 2007) with the BEAGLE library (Ayres et al., 2012) as a basis for further analyses. Sequence evolution was modelled using an HKY+gamma substitution model partitioned by first, second and third codon positions and intergenic regions, implemented with an uncorrelated lognormal molecular clock (Drummond, Pybus, Rambaut, Forsberg, & Rodrigo, 2003; Drummond & Suchard, 2010) and a Bayesian skyline model (Drummond, Rambaut, Shapiro, & Pybus, 2005). Five independent MCMC chains were run for 50 million steps, sampled every 50,000th and combined in logcombiner v1.8.1. Chains were inspected for stationarity and adequate mixing in tracer v1.6 (Rambaut & Drummond, 2014) and a 10% burn‐in discarded from each. The combined posterior tree distribution was subsampled to a set of 1,000 trees to provide an adequate sample of phylogenetic uncertainty. The resulting empirical tree set was used in all subsequent diffusion analyses to approximate phylogenetic uncertainty. A transition kernel was implemented to randomly sample from this tree distribution (Pagel, Meade, & Barker, 2004). A maximum‐likelihood phylogeny was also generated in raxml (Stamatakis et al., 2012), employing the GTRGAMMA model with 1,000 bootstrap replicates to showcase the genetic diversity in the data (Figure S2).

### 2.4 Measuring the local diffusion dynamic

Spatial diffusion was mapped on the posterior timescaled tree distribution estimated (as explained above) using the continuous phylogeography framework described by Lemey et al. (2010a,b). This enables the incorporation of precise geographic detail using a Brownian or relaxed random walk (RRW) process to estimate spatial diffusion, overcoming the need to force an (often unrealistic) discretized sampling scheme for analysis. A Brownian diffusion model, which assumes that the process does not vary over time, was tested alongside RRW models allowing dispersal rates to vary along branches according to gamma or lognormal prior distributions. Models were compared using marginal likelihood estimates obtained by path sampling (PS) and stepping‐stone (SS) sampling to choose the most appropriate diffusion model.

### 2.5 Measuring diffusion in attribute‐modified landscapes

Two main phylogeographic approaches were used to measure the effects of spatial heterogeneity on RABV diffusion. The methodological details of both are discussed below, and a comparative summary of each is provided in Table 2. Example XML files for each model are provided in Dataset S1.

**Table 2:** Comparison of phylogeographic approaches used to measure the effects of spatial heterogeneity on rabies virus diffusion

Approach	Defining RABV clusters	Phylogeographic trait	Extension to diffusion model	Measure of diffusion process	Incorporation of landscape attributes
Discrete‐MDS	Multidimensional scaling of RABV locations using a landscape resistance distance matrix, followed by *k*‐means clustering.	Landscape‐informed clusters	Markov jump counts to estimate numbers of migrations between clusters	1. Estimated migrations between clusters	Individually
				2. Phylogeny–trait association index	
GLM‐diffusion model	*k*‐means clustering of original RABV locations.	Geographic clusters (Euclidean distance)	GLM parameterization of the migration rate matrix using landscape predictors, that is, vectors of resistance distances between cluster centroids.	1. GLM inclusion probability formalized by Bayes factor support	Together
				2. Conditional effect size reflecting contribution of each attribute when included in the model.	

#### 2.5.1 Finding clusters for discrete diffusion models

Multidimensional scaling (MDS) was used to project RABV cases in two‐dimensional space representative of each landscape attribute in Table 1. MDS positions objects in an *N*‐dimensional space to represent information contained in a similarity or dissimilarity matrix. Here, the aim was to produce a rescaled spatial configuration of RABV cases representing the perceived proximity between cases according to landscape resistance. For each attribute, a matrix of Circuitscape resistance distances was used to inform MDS. For example, river resistance distances represent the expected impediment to RABV dispersal; therefore, cases separated by landscape cells with rivers present (i.e., high resistance) project further apart in MDS space (see Figure 3 for visualization).

For phylodynamic diffusion models, the rescaled RABV cases were divided into spatial clusters using a *k*‐means algorithm. To determine the number of clusters (*k*) needed to ideally represent the distribution of cases, various statistical methods were applied (see Methods S1). However, limited consensus between these methods meant an appropriate range (*k *=* *3–15) was instead used to explore the effect of spatial clustering and scale. Resulting spatial clusters for each *k* in the range were used to assign location states to each observed RABV case in a discrete phylogeographic analysis (Lemey, Rambaut, Drummond, & Suchard, 2009). Diffusion between locations was modelled using a nonreversible continuous‐time Markov chain (CTMC) process, which uses a *k* × *k* infinitesimal rate matrix Λ to describe migrations between *k*‐discrete locations. MCMC chains with a predefined tree space (the empirical tree set) were run for five million steps and sampled every 500. We refer to this approach as a discrete‐MDS phylogeographic diffusion model. Two measures were used to assess diffusion among clusters in comparison with a null model (i.e., in a uniform landscape):

1. *Migrations between clusters*. The number of expected migrations to explain diffusion throughout the inferred evolutionary history was estimated using Markov jump (MJ) counts (Minin & Suchard, 2008). A reduction in MJ counts (while keeping the number of clusters constant) across the phylogeny indicates a more parsimonious explanation for the observed spatial pattern.
2. *Phylogeny–trait association*. This was measured using a modified association index (AI) (Lemey et al., 2009; Wang, Donaldson, Brettle, Bell, & Simmonds, 2017), which reports the posterior distribution of association values relative to those obtained by randomizing the tip locations and represents the degree of spatial admixture. Low AI values represent strong phylogeny–trait association and low spatial admixture.

In summary, fewer MJ counts and stronger phylogeny–trait clustering than expected under a null model is indicative that the attribute has shaped population structure.

#### 2.5.2 Testing the relative contribution of attributes to the diffusion process

A generalized linear model (GLM) diffusion parameterization (Lemey et al., 2014) of the discrete diffusion model was applied to estimate the influence of landscape attributes on diffusion between discrete locations. Cases were partitioned into *k*‐discrete locations by MDS as explained above using a Euclidean distance matrix rather than the manipulated landscape in the previous approaches (Figures 3, S3, and S4). Landscape attributes for the GLM were constructed using Circuitscape resistance distances calculated between the centroids of each cluster (clusters shown in Figure S3, centroids in Figure S4) and were log‐transformed and standardized before their incorporation in the GLM. Pearson correlations between attributes were calculated (Table S2), and in cases where the correlation was greater than or equal to 0.9, a GLM with one of the correlated attributes removed was also tested to ensure it had no effect on the results obtained.

In the GLM approach, the migration rate matrix used to model diffusion is parameterized by a log linear function to incorporate a set of attributes on a log scale (Lemey et al., 2014). The relative contribution of each attribute *p* to the GLM is measured by a coefficient β, and a binary indicator δ determines the inclusion or exclusion of an individual attribute in the model. The indicator variables are estimated using Bayesian stochastic search variable selection (BSSVS). This estimates the posterior probability of all possible models including or excluding each attribute and so results in an estimate of the posterior inclusion probability for each attribute. A small prior probability was used on each predictor's inclusion reflecting a 50% prior probability of no predictor being included (Lemey et al., 2014). Bayes factors (BF) were calculated using δ estimates to assess the level of evidence against the null hypothesis, that is, the observed attribute inclusion (pp_p_) vs. the prior opinion for attribute inclusion (qp_p_).

To test the effect of cluster size, origin and destination cluster sizes (number of RABV sequences included per *k* location state in the phylogeographic analysis) were included in the GLM as separate attributes. Support for other attributes in addition to cluster size attributes suggests that analyses are robust to potential sampling biases.

A BF ≥ 3 was considered the threshold for sufficient support against the null hypothesis, which corresponds to pp_p_ being three times more likely than qp_p_ (when an attribute is included 50% of the time). MCMC chains were run for five million steps and sampled every 500.

### 2.6 Overall evidence

To summarize results overall, each attribute was ranked according to the strength of evidence from each measure of the diffusion process. Scores for each measure were calculated and summed as follows:

1. Results from non‐GLM based measures of diffusion, that is, with *k* attribute‐defined clusters, were condensed to the larger spatial scales tested, *k *=* *3–6, as this appeared to be the most relevant spatial scale to test landscape effects. Each attribute was ranked in ascending order according to (i) the sum of the mean number of migrations and (ii) the sum of the mean AI ratio at each *k‐*level.
2. Generalized linear model results were ranked according to the strength of Bayes factor support in descending order. An overall BF value per attribute was calculated via the sum of significant BF results across *k*‐values with the highest value ranked first. Attributes with no significant results were equally scored last.

## 3 RESULTS

### 3.1 Local transmission dynamics

**Figure 2:**
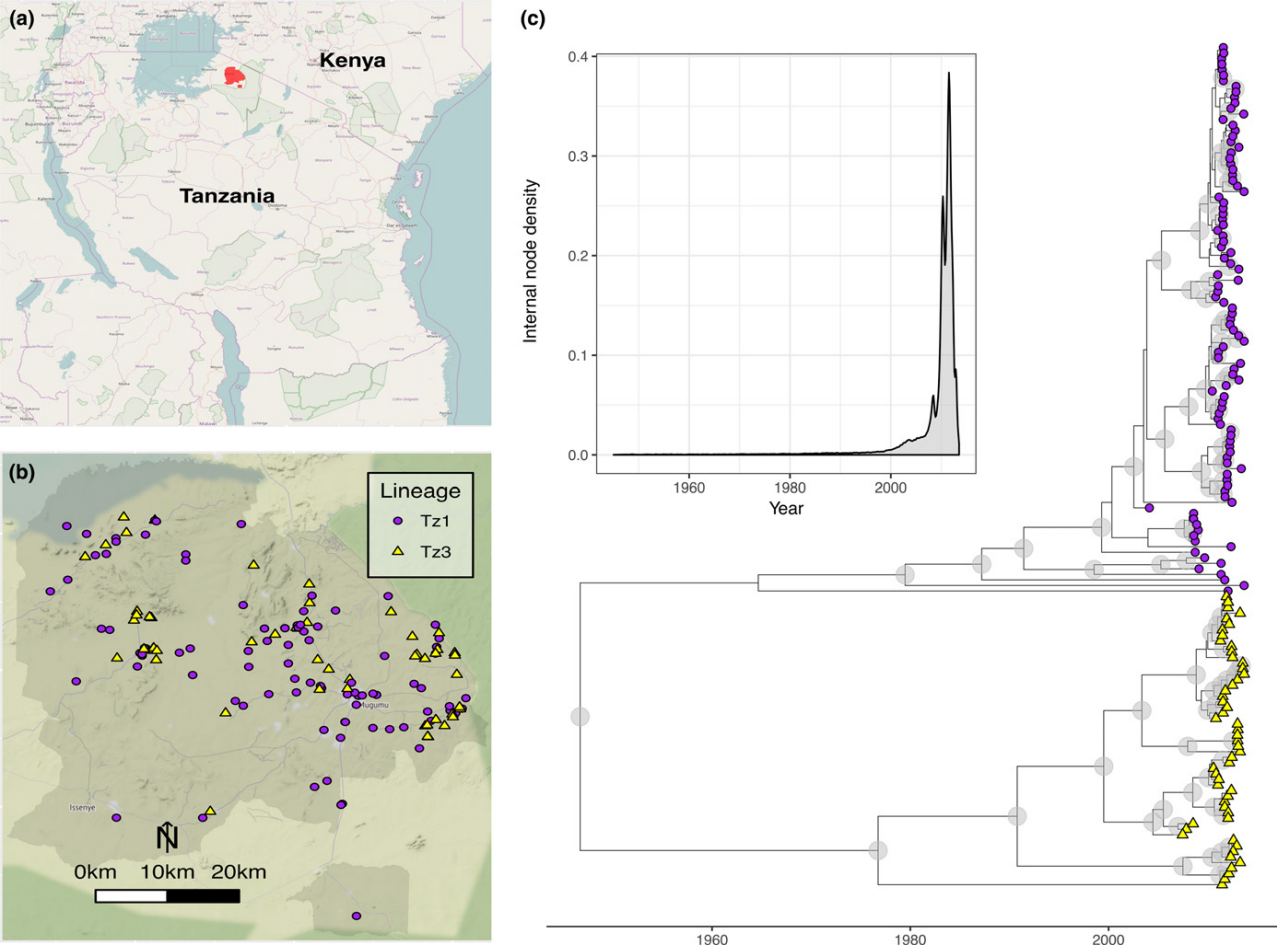
The spatial location and phylogenetic structure of 152 sequenced rabies viruses sampled from 2004 to 2013 within the Serengeti district, Tanzania. (a) The Serengeti district (red polygon) within Tanzania; (b) locations of sequenced rabies cases within the Serengeti district (grey polygon) with underlying topography (map tiles by Stamen Design, under CC BY 3.0. Data by OpenStreetMap, under ODbL.) and administrative boundaries from http://www.nbs.go.tz; (c) timescaled maximum clade credibility tree from a Bayesian phylogenetic reconstruction of whole‐genome sequences, with node posterior support >0.9 indicated by blue circles. The inset shows node density through time for the posterior set of trees, with >90% nodes occurring in the last 10 years. Maps drawn using R packages OpenStreetMap (Fellows & Stotz, 2016) ggmap (Kahle & Wickham, 2013) and maptools (Lewin‐Koh et al., 2012)

A set of timescaled trees for full‐genome RABV sampled in the Serengeti district (Figure 2) was estimated using BEAST. The coordinates of internal nodes were mapped to this empirical tree set according to a continuous diffusion process, quantifying the rate and variation of rabies spread. A RRW model with branch diffusion rates drawn from a gamma distribution provided the best fit (model selection in Table S1) on the empirical tree set. The mean rate of RABV spread was estimated at 4.46 km/year (95% HPD: 3.22–5.88), similar to estimates for endemic wildlife RABV spread (Biek, Henderson, Waller, Rupprecht, & Real, 2007; Lemey et al., 2010a,b), but around four times lower than estimates for dog RABV spread in North Africa (Talbi et al., 2010). There was considerable variation in the diffusion rate among branches, indicated by a large coefficient of variation (*M* = 3.10) for rates drawn from the gamma hyperdistribution in the RRW diffusion model. Two major lineages were identified (in agreement with a previous study [Brunker et al., 2015]), which cocirculated throughout the sampled period (Figure 2). In addition, this analysis yielded a mean evolutionary rate of 2.67 × 10^−4^ substitutions/site/year in line with dog RABV estimates for nucleoprotein, glycoprotein and whole‐genome evolution elsewhere (Ahmed et al., 2015; Bourhy et al., 2008, 2016; Talbi et al., 2010).

### 3.2 The effect of landscape heterogeneity on RABV movement

**Figure 3:**
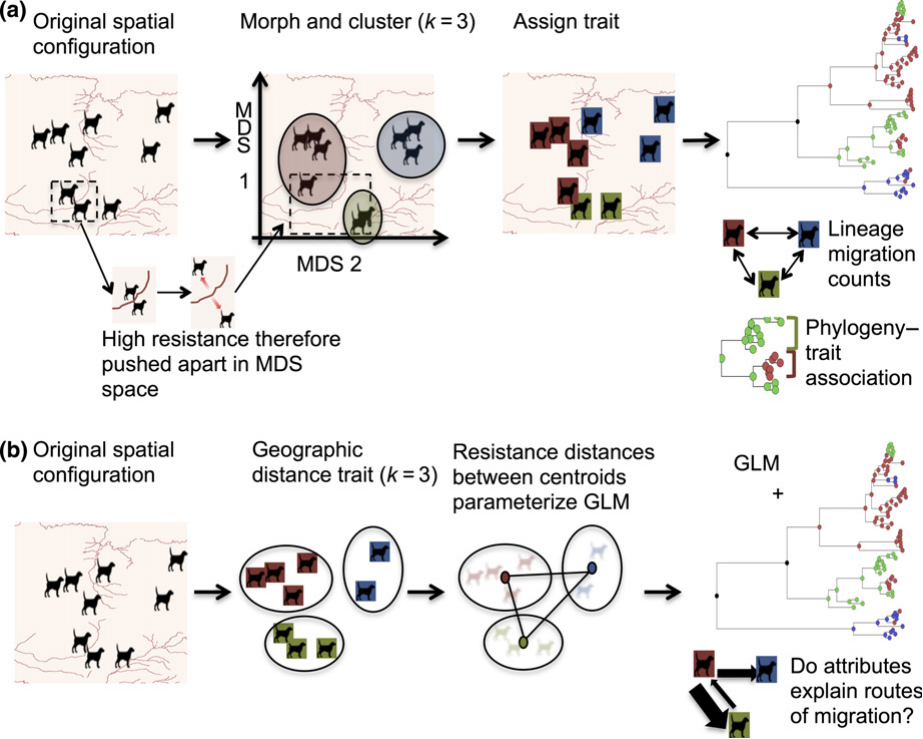
Using resistance distances to incorporate landscape heterogeneity into phylogeographic frameworks. Illustration of resistance surfaces assuming rivers (dark red) acts as barriers to RABV spread. Two approaches are used to incorporate resistances in discrete phylogeographic reconstructions: (a) locations of sequenced rabies cases are morphed in space using multidimensional scaling (MDS) and clustered according to a *k*‐means partitioning scheme (*k *=* *3 shown). MDS cluster information is used to assign traits in a discrete trait phylogeographic reconstruction measuring viral lineage migrations and phylogeny–trait association; (b) locations are clustered according to geographic distances using *k*‐means partitioning and resistance distances between cluster centroids are used to parameterize a GLM extension of discrete phylogeographic diffusion. Bayesian model averaging is used to identify significant predictors of viral spread between centroids

Landscape heterogeneities (Table 1 and Figure 1) were incorporated into discrete phylogeographic diffusion models by rescaling spatial locations according to landscape‐informed resistance measures and subsequent clustering of the rescaled locations (Figure 3a). The relative contribution of each predictor was further estimated using a GLM approach (Lemey et al., 2014) parameterized by resistance measures (Figure 2b).

**Figure 4:**
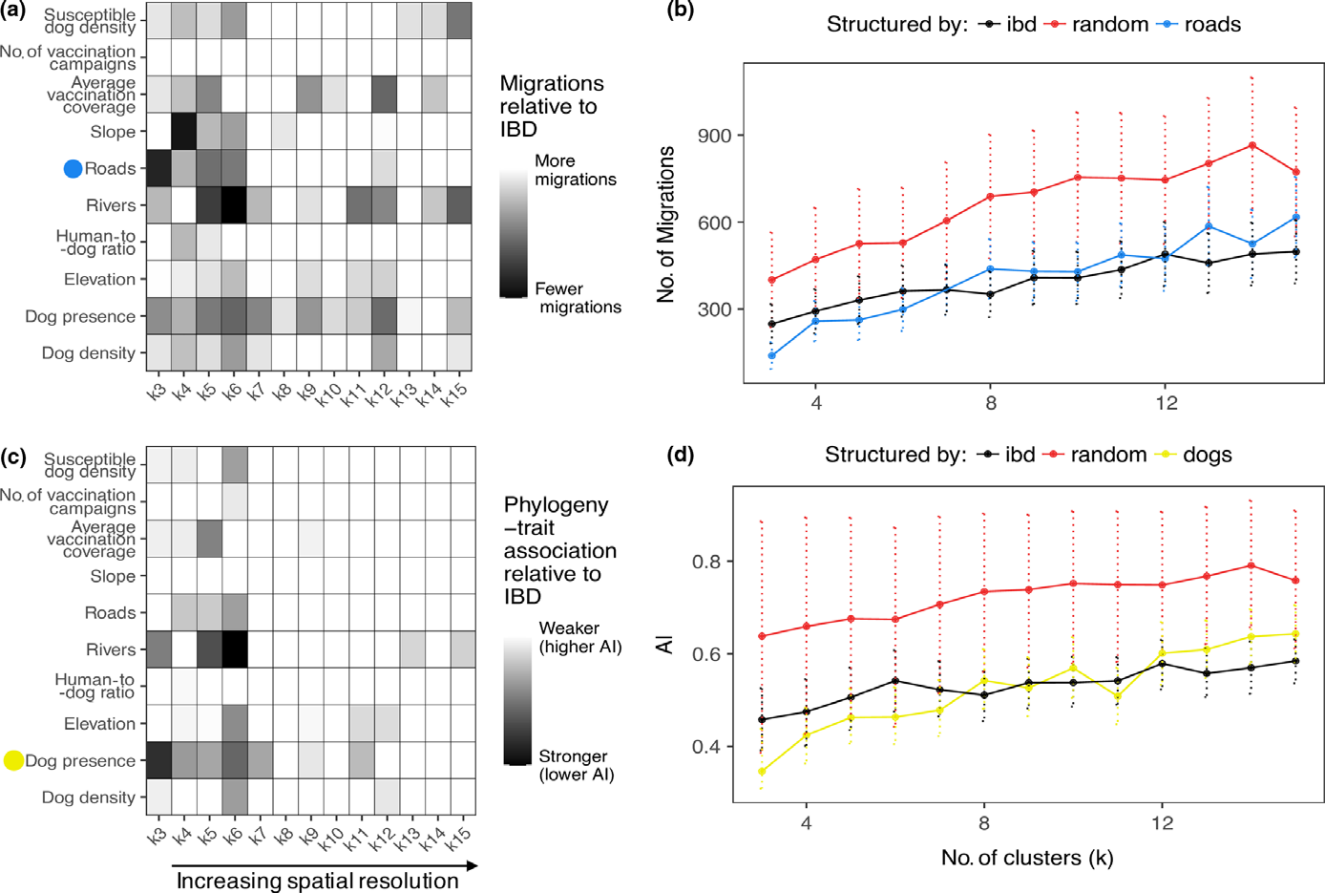
Summarized results from discrete‐MDS phylogeographic models using landscape‐informed spatial clusters for reconstructed RABV movement in Serengeti district. A number of spatial scales were examined by subjecting RABV cases (*n* = 152) to different levels of partitioning (*k*), ranging from 3 to 15 clusters. (a) A heatmap representing the reduction in estimated viral lineage migrations relative to a null model (where only isolation by distance (IBD) was used to inform spatial clustering) at each *k* (horizontal axis) when each landscape attribute (vertical axis) informed the configuration of clusters. White cells represent no reduction or an increase in migrations (i.e., the null model was better), whereas shaded cells represent fewer migrations between attribute‐informed clusters compared to the null model (i.e., the attribute‐informed model was better). (b) The number of inferred migrations at each spatial scale when clusters were assigned randomly, according to IBD, or by roads (which showed the largest reduction in migrations relative to IBD at *k *=* *3–6). (c) A heatmap representing the improvement in phylogeny–trait association according to an association index, AI, for landscape‐informed clusters relative to IBD‐informed clusters, with smaller AI values indicating stronger associations. (d) The inferred AI at each spatial scale when clusters were assigned randomly, according to IBD, or using dog presence (which had the strongest phylogeny–trait association at smaller values of *k*)

Rabies virus movement was assessed by assigning samples to discrete spatial clusters defined by landscape attributes (Figure 3a). As the appropriate scale for analysis was not known a priori, the number of clusters (*k*) was varied from 3 to 15 for each attribute. Two measures of diffusion were assessed for each landscape attribute: the estimated number of viral lineage migrations according to Markov jump (MJ) counts and a phylogeny–trait association index (AI), with strength of support expressed relative to IBD, as a null model. Clusters structured according to landscape attributes (including IBD) always exhibited fewer migrations and higher phylogeny–trait association than randomized data (Figure 4), consistent with these attribute‐transformed landscapes providing an improved measure of viral diffusion. A large number of migration events were recorded overall, indicating considerable local movement across this landscape. Results varied according to *k,* but most attributes were consistently better at explaining viral diffusion than IBD at larger spatial scales (*k *=* *3–6) as illustrated for roads (Figure 4b) and the presence of dogs (Figure 4d).

The AI was calculated to assess the degree of phylogeny–trait association, that is, attribute‐defined structure according to the number of clusters, *k*. There was some congruence between the measures; that is, structure tended to be stronger when there was also a large reduction in the number of lineage migrations in particular for dog presence, rivers and to some extent roads. However, migration count were generally more sensitive than AI results (Figure 4).

### 3.3 Relative contribution of attributes to RABV movement

A GLM approach (Figure 3b) within a Bayesian framework was used to identify landscape attributes driving the spread of RABV in the Serengeti district. Geographic clusters (based on standard geographic distances) were defined using the same range of *k* as before, and GLMs were parameterized using resistance distances between the centroids of these clusters. Total dog density, susceptible dog density, vaccination coverage and campaigns, human:dog ratios (HDR) and roads all had no discernible support at any spatial scale using this approach. The effect of cluster size (note: this is the number of RABV cases per cluster, which is different from the number of clusters, *k*) was tested by its inclusion as an attribute in the GLM with the purpose of absorbing any potential adverse effect of sampling bias. This offers more credibility on the effect of landscape attributes, which otherwise might owe their support to correlations with sample size. However, we found cluster size had little effect on the main results except to eliminate some attributes with borderline significance (according to a threshold of BF > 3). When cluster size was included, dog presence, elevation, rivers and slope were the only attributes that surpassed the BF threshold at certain spatial scales, indicating an influence on RABV movement (Table 3).

**Table 3:** Landscape attributes influencing the dispersal of RABV in the Serengeti district, Tanzania. Bayes factor support and conditional effect sizes from GLM‐diffusion models implemented in BEAST are shown for BF significance >3 at different spatial discretizations (number of clusters, *k*)

Landscape attribute	*k*	Inclusion probability	Conditional effect size	Bayes factor
Dog presence	7	0.82	−1.11 (−1.76, −0.56)	76.4
	9	0.2	−0.8 (−1.28, −0.33)	4.17
	12	0.16	−0.86 (−1.36, −0.4)	3.15
	13	0.2	−0.84 (−1.34, −0.38)	4.13
Elevation	12	0.46	−0.9 (−1.35, −0.47)	14.2
	13	0.5	−0.87 (−1.34, −0.41)	16.95
	14	0.58	−0.94 (−1.5, −0.44)	23.17
	15	0.16	−0.83 (−1.37, −0.3)	3.31
River	12	0.32	−0.78 (−1.16, −0.42)	7.98
	15	0.49	−0.73 (−1.06, −0.39)	15.88
Slope	15	0.16	−0.62 (−0.98, −0.26)	3.26

Results were scale‐dependent, but in general, significant effects were more often found when a greater number of centroids was used to build the GLM migration matrices. All significant attributes had a negative effect size, consistent with lower rates of RABV movement as the effective resistance of the attribute increased. For facilitators, for example, roads, this means that an increased presence (lower resistance) results in more RABV movement. For barriers, for example, rivers, an increased presence (higher resistance) results in less RABV movement. The strongest effect was found when dog presence was included in a model with *k *=* *7 (BF = 76.4, with a mean negative conditional effect size of 1.11). Dog presence also had an impact at larger *k* (*k *=* *9, 12, 13), that is, at higher resolution. Elevation was supported at four scales (*k* = 12–15) with an estimated negative effect size ranging from −0.83 to −0.94, indicating less RABV movement at higher elevations. Rivers also had reasonable support at two spatial scales (*k *=* *12 & 15), again with a negative effect size indicating slower diffusion across rivers. In instances where attributes were highly correlated (Table S2), a simplified GLM with the removal of one attribute was performed and in all cases showed equivalent results to the full GLM (results not shown).

### 3.4 Overall results

To assess the overall evidence for landscape attributes influencing viral movement, a scoring system was used to rank each attribute from 1 to 10, with 1 being the most supported (Table 4). Results for non‐GLM‐based measures were limited to scales from *k* = 3 to *k* = 6 as results became less discernible from the null IBD model at *k* > 6 (Figure 4). Dog presence showed strong and consistent levels of support in each measure of the diffusion process, indicating that the distribution of the dog population is the most important determinant of RABV transmission. Elevation also ranked highly, which can be regarded as an indicator of host distribution given that human settlements (and therefore dogs) are less common at higher elevations (Cohen & Small, 1998). There was considerable support for the impact of physical attributes on host movement with rivers as barriers and roads as facilitators, while slope performed reasonably well in some measures. Total dog density had limited effect on measures of RABV movement, but susceptible dog density was scored marginally higher (Table 4). There was some evidence that vaccination measures limited spread, with average vaccination coverage and the susceptible dog density both performing better than the null IBD model. However, the consistency of vaccination campaigns over a 10‐year period had no apparent effect on RABV movement, making no improvement on the null model of IBD or generating any significant results (BF > 3) in the GLM.

**Table 4:** Overall support for individual landscape attributes as predictors of RABV spread in the Serengeti district, Tanzania

Attribute	Overall rank	Overall score	Lineage migration counts	Association index	GLM Bayes factor
Dog presence	1	5	3	1	1^a^
Rivers	2	6	2	2	2^a^
Roads	3	12	1	6	=5
Elevation	4	13	7	3	3^a^
Average vaccination coverage	5	15	5	5	=5
Susceptible dog density	6	17	8	4	=5
Slope	=7	18	4	10	4^a^
Dog density	=7	18	6	7^+^	=5
Human‐to‐dog ratio	9	22	9	8^+^	=5
No. of vaccination campaigns	10	24	10^+^	9^b^	=5

=, equal score/rank for attributes.

a Significant effect in GLMs according to Bayes factor > 3.

b Measure did not improve on the null model.

## 4 DISCUSSION

Integrative spatial analyses drawing from phylogeography and landscape ecology provide an exciting new avenue to explore infectious disease dynamics (Lemey et al., 2014; Trovão et al., 2015). By combining isolation‐by‐resistance (IBD) theory from landscape ecology with powerful Bayesian phylogeographic analyses, we identified drivers of endemic RABV spread beyond IBD and demonstrated scale‐dependent landscape effects on transmission. Once IBD effects were accounted for, we identified the distribution of dogs as the most important predictor of RABV spread, but did not find evidence of dog density effects. This supports assertions that RABV is maintained primarily in domestic dog populations rather than wildlife and that transmission does not depend on dog density (Hampson et al., 2009; Morters et al., 2013). Our results demonstrate the potential for both fundamental and applied insight into the local drivers of endemic RABV spread, but also highlight the need for further methodological development to understand how transmission processes scale from the individual to the landscape.

In line with our understanding of pathogen transmission, distance was by far the most important attribute in explaining local RABV spread. Once the two major cocirculating lineages were differentiated, phylogenetic signatures revealed that most cases nearby in space and time were highly related. Indeed, connectivity determined by IBD (our null model) consistently explained more variation in viral diffusion models than a randomized spatial structure (Figure 3). Our estimated mean diffusion rate for RABV of 4.46 km/year is 4–8 times lower than dog RABV diffusion estimates from three North African countries (Talbi et al., 2010), but higher than the rate observed in a densely populated Central African city (Bourhy et al., 2016). Our estimate is very close to that of endemic wildlife RABV (Biek et al., 2007; Lemey et al., 2010a,b) where natural host movement is the main mode of spread, suggesting that persistence of endemic dog RABV in the Serengeti is maintained by the same mechanism. However, the diffusion model showed considerable variation in the diffusion rate among branches, which suggests a potential role for landscape heterogeneities in explaining variation which comprises both rabid dog movement and human‐mediated translocations.

Overall, we showed that local presence of dogs is the most important predictor of RABV transmission in the Serengeti district, confirming the role of dogs as the main reservoir host and not wildlife (Lembo et al., 2007). The effects of dog presence on RABV diffusion were evident across multiple spatial scales, indicating that uninhabited areas limit RABV movement over a range of spatial distances. Dog home ranges typically do not extend beyond a 1 km^2^ radius (Hampson et al., 2009; Woodroffe & Donnelly, 2011). RABV transmission beyond this may require inhabited corridors that direct dog movement and/or support chains of transmission. Given the strong association between humans and dogs (Figure S1), the presence of humans should be a reasonable proxy (and more accessible resource) for dog presence that could inform models of RABV spread.

The impact of physical barriers or conduits was most evident at larger spatial scales that effectively divided the landscape into three to six subpopulations. Roads increased RABV movement, as in North Africa (Talbi et al., 2010), and resulted in the largest reduction in viral lineage migrations at larger scales, implying that the furthest dispersal of RABV was associated with roads, consistent with human‐mediated movement of dogs. However, roads typically circumvent physical barriers and uninhabited land and thus could also reflect the accessibility of the landscape to unaided dog movement. Either way, roads represent routes of RABV dissemination and indicate the increasing importance of landscape connectivity as spatial scale surpasses the limits of natural dog movements (~1 km). It could be argued that the effect of roads may be driven by surveillance bias if rabid dogs are more likely to be detected and sampled near roads. If this were the case, we would have expected to see a positive effect of high human‐to‐dog ratios. However, no such effect was observed.

Rivers reduce the dispersal of wildlife rabies (Bourhy et al., 1999; Rees et al., 2008; Wheeler & Waller, 2008), and our results suggest that rivers similarly impede movement of rabid dogs, even at very local scales. Deployment of vaccines behind rivers could therefore be beneficial for eliminating dog rabies as recommended for control of wildlife rabies (Russell et al., 2006). More generally, these results suggest a role for landscape attributes mediating metapopulation dynamics (introductions and extinctions) that contribute to RABV persistence (Beyer et al., 2011; Bourhy et al., 2016).

Our results provide insights regarding the value of control measures. Specifically, our finding that village‐level vaccination coverage reduced RABV dispersal is encouraging, particularly given the crudeness of the measurement used (coverages averaged over a 10‐year period). WHO recommends vaccination coverage should exceed 70% (WHO, 2013), but we found that lower coverage still impedes dog rabies spread. However, the relationship between vaccination coverage and disease appears complex (Beyer et al., 2011), and we did not detect any association with numbers of vaccination campaigns (a measure of the consistency of vaccination over time). Sequenced genomes represent approximately 10% of identified rabies cases during this period; therefore, direct measures of incidence are likely to yield more insight on the impacts of vaccination. Total dog density did not contribute to RABV movement, which substantiates evidence that rabies transmission is not density dependent (Hampson et al., 2009; Morters et al., 2013). Susceptible dog density, however, which accounted for vaccination, was superior to total density as an explanatory variable, but still had limited effect on diffusion. These results add to the now substantial evidence base that mass vaccination of dogs, not population reduction, is required for effective rabies control (Hampson et al., 2009; Morters et al., 2013).

The effect of landscape attributes may be scale‐dependent; therefore, efforts were made to find the most representative discretization (*k*) for each attribute (Methods S1). However, different methods did not converge on the same optimum *k*. Challenges associated with geographic partitioning in phylodynamic models have previously been noted, including scale‐dependent outcomes and sampling‐bias effects (Lemey et al., 2014). Choosing an appropriate partitioning scheme based on a biological hypothesis or testing a range of partitioning schemes is therefore an important consideration. We tested the effect of cluster size by including it as a covariate in GLM‐diffusion models, with negligible effects on results. This and the consistency of our results across similar spatial aggregations implies that observed effects on diffusion are robust. A number of attributes showed consistently strong results using fewer partitions but diminished effects at higher resolutions in the discrete‐MDS phylogeography approach.

The GLM‐diffusion model supported the role of landscape attributes at smaller scales (*k* centroids >6), with dog presence, elevation, rivers and slope all identified as significant predictors of diffusion (median *k *=* *13, Table 3). Using cluster centroids means that finely resolved heterogeneity is lost, with less detail available to effectively characterize the landscape at large‐scale discretizations. The sensitivity of this approach therefore depends on the scale of analysis, with biological knowledge required to assess whether cluster centroids are expected to capture landscape heterogeneities.

An attractive property of the GLM‐diffusion approach is the ability to assess the relative contribution of different attributes. However, highly correlated resistance distances such as total and susceptible dog density present a problem as they potentially explain the same variation. Simplified GLMs were performed to verify results from the full model that included all attributes. However, even when resistances are correlated, one might offer a marginally better fit (Talbi et al., 2010). For example, the observation that the susceptible dog density provides better explanatory power than total density fits with expectations regarding the effect of vaccination. As many of the attributes tested were correlated due to shared underlying IBD structure, such subtle differences may be necessary to extract the most meaningful predictors for pathogen transmission at the landscape scale. A more powerful approach would be to produce a multivariate surface representing the combined attributes affecting diffusion. This introduces further considerations, including identifying collinearity between attributes and comparative resistance values of attributes, but should be an aim for future studies.

We capitalize on the use of resistance surfaces to represent landscape attributes. While synthesizing landscape information in this way is useful, determining appropriate resistance values is a common methodological challenge in landscape ecology for which there is currently little consensus (Beier, Majka, & Spencer, 2008; Beier, Spencer, Baldwin, & Mcrae, 2011; Spear, Balkenhol, Fortin, McRae, & Scribner, 2010; Zeller, McGarigal, & Whiteley, 2012). Ideally, resistances should be parameterized from empirical data, but expert opinion is often used when such data are unavailable (Beier et al., 2008). We assumed linear relationships between continuous variables and resistance, such as elevation or vaccination, but nonlinear relationships could be more informative if they for instance capture threshold effects (Spear et al., 2010). Although not ideal, our parameterization scales with biologically meaningful quantities and reflects the relative effects of attributes on diffusion, which is more important than the choice of absolute resistance values (McRae, 2006). However, it may be advisable to check for the effect of resistance value parameterization by repeating analyses with a different parameter values and testing the attribute as both a facilitator and a barrier to viral spread, as performed by Dellicour et al. (2017). Some landscape attributes, however, may not be well represented by resistance surfaces, particularly those that are heterogeneous through time. For instance, we summarized vaccination coverage over a 10‐year window discarding known and potentially important temporal fluctuations, which likely limited predictive power. A recently developed application to relax the time‐homogeneity assumption in phylogeographic reconstructions has demonstrated seasonal effects on the dispersal of influenza H3N2 and suggests that further developments may enable incorporation of temporal variation (Bielejec, Lemey, Baele, Rambaut, & Suchard, 2014).

## 5 CONCLUSION

Increasing availability of genetic and spatially and temporally resolved data provide opportunities to better understand transmission mechanisms in complex host–pathogen systems. Using an integrative Bayesian phylogeographic framework, we quantified the effect of landscape heterogeneity on the transmission and spread of endemic RABV. Given a number of outstanding issues, including the parameterization of resistance surfaces, efforts to directly apply these results, for example, to inform control efforts, should proceed with caution. Nonetheless, results suggest that key landscape attributes could be exploited to limit RABV spread. Importantly, the finding that the distribution of dogs but not their density predicts RABV spread supports mass dog vaccination as the mainstay of effective rabies control even in wildlife‐rich communities such as Serengeti and reinforces the conclusion that culling of dogs should not be used to control rabies. Moreover, by exploiting landscape heterogeneities during the roll‐out and scaling up of campaigns, vaccination programmes could be strengthened. From a methodological perspective, this study demonstrates the potential of phylogeographic techniques to identify important landscape attributes governing pathogen dispersal in endemic settings.

## DATA ACCESSIBILITY

New DNA sequences submitted with this paper: Genbank Accession nos. KY210220–KY210311. Previously published DNA sequences: Genbank Accession nos. KR534217–KR534220; KR534228‐KR534238; KR534244‐KR534254; KR534256; KR906734; KR906737‐KR906738; KR906740; KR906742; KR906755‐KR906756; KR906767‐KR906792.

## AUTHOR CONTRIBUTIONS

K.B., P.L., K.H. and R.B. were involved in study design and concept. A.L. and C.N. coordinated regional field sample collections. A.R.F. and D.A.M. facilitated laboratory work and provided molecular expertise. K.B. performed molecular work, sequencing, bioinformatics and analysis. P.L. provided support and training in bioinformatic analysis. K.B. wrote the manuscript with significant contributions from K.H. and R.B. All authors viewed and revised final manuscript.

## ETHICS STATEMENT

This research was approved by the Institutional Review Board of Ifakara Health Institute, Tanzania National Parks, the Tanzania Wildlife Research Institute, the Tanzania Commission for Science and Technology and the Medical Research Coordinating Committee of the National Institute for Medical Research of Tanzania (NIMR/HQ/R.8a/Vol.IX/946) and the Ministry of Livestock Development and Fisheries including permits for sample collection (VIC/AR/ZIS/4376).
