# Supplementary material for: Landscape attributes governing local transmission of an endemic zoonosis: Rabies virus in domestic dogs
Source: Mol Ecol. 2018 Jan 29;27(3):773–88. doi: 10.1111/mec.14470 (PMC5900915; doi:10.1111/mec.14470)

MOLECULAR ECOLOGY

Supplemental Information for:

.andscape attributes governing local transmission of an endemic zoonotic virus: rabies in domestic dogs.

Kirstyn Brunker, Philippe Lemey, Denise A. Marston, Anthony R. Fooks, Ahmed Lugelo, Chanasa Ngeleja, Katie Hampson, Roman Biek

Table of Contents:

**Methods S1**	Page 1
**S1 Table**	Page 6
**S2 Table**	Page 7
**S3 Table**	Page 9
**S1 Fig**	Page 30
**S2 Fig**	Page 31
**S3 Fig**	Page 32
**S4 Fig**	Page 33

METHODS S1

454 pyrosequencing

Whole genome sequences for five samples (see Table S1) were generated via 454 pyrosequencing protocols. DNA for three samples was prepared according to the depletion method detailed in the main methods and sent to the Animal & Plant Health Agency (APHA) sequencing facility for the preparation of sequencing libraries and 454 pyrosequencing. The other two samples were prepared according to a PCR amplicon approach as follows. TRIzol-extracted viral RNA was reverse transcribed to cDNA using the primer RABV_Tzdg.p1f (5' ACGCTTAACAACAAAATCAGAG 3') at a concentration of 2pmol/µl and Superscript III reverse transcriptase (Invitrogen) in a total volume of 20µl, as per manufacturer's instructions. An in house set of 26 short, tagged, overlapping primer pairs spanning the entire RABV genome was used to obtain PCR products with 1µl of cDNA and

KOD hot start DNA polymerase kit as per the manufacturer's protocol (Novagen). The following cycling parameters were used: 1 hold at 95 °C for 2 mins, 35 cycles at 95 °C for 20 s, 50-60°C (dependent on the optimized temperature for each primer pair) for 20 s, 70 °C for 20 s and a final hold at 70 °C for 10 min. Products were pooled for each sample with libraries and sequencing completed by the APHA sequencing facility.

Construction of resistance surfaces

Landscape attributes were characterized as surface models by assigning cell values to represent the assumed facilitating or impeding impact of a predictor on RABV diffusion. Resistance surfaces for each attribute were formatted as follows:

i) Dog density

Dog density estimates were taken from a household census conducted in the Serengeti District in 2014-2015, which provided the GPS location and dog count for each household. The density point pattern was smoothed across the default raster grid using an isotropic Gaussian smoothing kernel with sigma=500 using the R package spatstat (1) with cell values expressing the estimated intensity values. As dog density is assumed to facilitate RABV diffusion, the reciprocal of these values was used as a resistance value in each cell. Serengeti National Park (SNP) areas were assigned a low resistance value, equivalent to 1 dog per km^2 to reflect the low dog density in this area. Cells outside the district (accounting for ~12% of all cells) were assigned random values from the Serengeti density data.

ii) Dog presence

Dog census data as described above was used to assign a presence or absence value to 100m grid cells based on the exact locations of dogs. Dog presence was assigned a low resistance value of 0.1 to represent conductive "corridors" of movement through normal landscape, whereas absence was the null IBD value of 1 to represent higher resistance to dog movement outside connected corridors.

iii) Elevation and slope

A digital elevation model covering the landscape was converted to raster format and elevation values used directly as resistance values. Slope values were calculated from the digital elevation model using the SDMTools package (2) in R. All slope values were increased by a value of 1 to ensure comparison to a null IBD surface was possible. The resolution of the digital elevation model was not consistent across the extent of the landscape as some areas have been mapped in more detail, which resulted in some finer grained areas in raster grids.

iv) Human to dog ratio (HDR)

Household census data was used to estimate the human to dog ratio per village, which was assigned directly as a resistance value in each cell.

v) Major roads and rivers

Shapefiles of major roads and rivers in the study area were converted to raster grids (one for roads, one for rivers) with the defined spatial extent and resolution. Cells containing a road feature were assigned a low resistance value of 0.001 to represent increased diffusion along roads relative to uniform landscape, all other cells were assigned a value of 1. River cells were assigned high resistance values of 1000 to reflect their influence as a barrier to diffusion.

vi) Average vaccination coverage

Mass dog vaccination campaigns have been undertaken in the Serengeti District since 2002, with varying annual coverage across villages. We used an average annual % vaccination coverage per village across the 11-year period from 2002 up to 2013 and assigned values to grid cells as resistance values [range: 6.43-100]. Rabies appears to have been locally eliminated in the SNP and there is a requirement for all dogs to be vaccinated within the park boundaries, therefore SNP cells were assigned the highest resistance value of 100. Cells outside the district and SNP were assigned the minimum observed average vaccination coverage of 6.43% as there is no formal vaccination initiative in these areas and therefore coverage is assumed to be low.

vi) Susceptible dog density

The dog density estimates were depleted according to the averaged vaccination coverage described above to produce a resistance surface representative of the susceptible host population density.

MOLECULAR ECOLOGY

vii) Number of vaccination campaigns

The number of vaccination campaigns conducted across the sampled time period 2004-2013 was summed for each village and assigned to each grid cell. Note some villages experienced return visits when turnout was less than expected, these were counted as separate campaigns if a coverage of at least 10% was achieved.

Determining an optimum location state partitioning scheme

Rabies cases projected in MDS attribute-defined space were partitioned according to varying levels of spatial aggregation using a *k*-means algorithm. The following methods were used to obtain an optimum range of *k*-values to determine a partitioning scheme:

i) Elbow method: the point of maximum curvature in a plot of number of clusters versus within-group sum of squares;

ii) Partitioning around medoids: using the optimum average silhouette to estimate the number of clusters, R package: fpc (3)

iii) Model-based clustering: chooses the optimal model and number of clusters according to Bayesian Information Criterion for expectation-maximisation, R package:mclust (4)

iv) Affinity propagation: a clustering algorithm that takes a pairwise similarity matrix and simultaneously considers all data points as potential cluster centres. The algorithm finds an optimum set of clusters that maximises the total similarity between data points and their cluster centres by an iterative process (5), R package: apcluster (6);

v) Gap statistic: a statistical procedure to formalise the "elbow" method by comparing the change in within-cluster dispersion to a reference null distribution (7), R package: cluster (8);

vi) R package NbClust: provides 30 indices to determine the number of clusters (9).

1. Baddeley A, Turner R. spatstat: An R Package for Analyzing Spatial Point Patterns. J Stat Softw [Internet]. 2005;12(6):1–42. Available from: http://citeseerx.ist.psu.edu/viewdoc/summary?doi=10.1.1.126.8464

2. VanDerWal J, Falconi L, Januchowski, Stephanie Shoo L, Storlie C. SDMTools: Species Distribution Modelling

Tools: Tools for processing data associated with species distribution modelling exercises. 2014.

3. Hennig C. fpc: Flexible procedures for clustering [Internet]. 2014. Available from: http://cran.r-project.org/package=fpc

4. Fraley C, Raftery AE. Model-Based Clustering, Discriminant Analysis, and Density Estimation. Journal of the American Statistical Association. 2002. p. 611–31.

5. Frey BJ, Dueck D. Clustering by Passing Messages Between Data Points. Science (80-). 2007;315(5814):972–6.

6. Bodenhofer U, Kothmeier A, Hochreiter S. Apcluster: An R package for affinity propagation clustering. Bioinformatics. 2011;27(17):2463–4.

7. Tibshirani R, Walther G, Hastie T. Estimating the number of clusters in a data set via the gap statistic. J R Stat Soc Ser B (Statistical Methodol [Internet]. 2001;63:411–23. Available from: http://onlinelibrary.wiley.com/doi/10.1111/1467-9868.00293/abstract

8. Maechler M, Rousseeuw P, Struyf A, Hubert M, Hornik K. Cluster Analysis Basics and Extensions. R package version 2.0.1 [Internet]. CRAN. 2015. Available from: http://cran.r-project.org/web/packages/cluster/index.html

9. Charrad M, Ghazzali N, Boiteau V, Niknafs A. NbClust: An R Package for Determining the Relevant Number of Clusters in a Data Set. J Stat Softw. 2014;61(6):1–36.

S1 Table. Model selection results of the continuous phylogeography diffusion model for Tanzania rabies virus dataset. Shown are marginal likelihood estimates using path sampling and stepping stone sampling in BEAST, with the best model in bold.

Diffusion model	Path Sampling	Stepping Stone
Homogeneous	-26674.93	-26679.87
**Gamma**	**-26661.45**	**-26666.37**
Lognormal	-26683.68	-26688.12

MOLECULAR ECOLOGY

S2 Table. Pearson's correlation coefficients between paired resistance distance matrices for landscape attributes used to parameterize GLM diffusion models estimating the rabies virus movement in the Serengeti District. Pairwise resistance distance matrices were constructed for *k*=5 to 15 centroids. Correlations greater than 0.9 are highlighted in large bold text.

Pairwise attribute combinations	k5	k6	k7	k8	k9	k10	k11	k12	k13	k14	k15
Campaigns,Dog density	0.55	0.85	0.58	0.45	0.56	0.51	0.70	0.54	0.35	0.30	0.39
Campaigns,Dog presence	0.58	0.65	0.66	0.13	0.30	0.12	0.08	0.57	0.26	0.34	0.41
Campaigns,Elevation	0.53	0.50	0.67	0.21	0.36	-0.21	-0.12	0.58	-0.11	0.44	0.23
Campaigns,HDR	0.48	0.42	-0.05	0.63	0.70	-0.53	-0.05	0.62	-0.32	0.17	0.74
Campaigns,Rivers	-0.29	0.10	0.02	-0.13	0.07	-0.22	-0.27	0.15	0.12	0.30	0.00
Campaigns,Roads	0.87	0.74	0.60	0.57	0.56	0.27	0.17	0.63	0.62	0.28	0.02
Campaigns,Slope	-0.01	0.15	-0.15	-0.28	-0.31	-0.67	-0.62	-0.12	-0.27	-0.10	-0.51
Campaigns,Susceptibles	0.52	0.84	0.66	0.53	0.67	0.46	0.68	0.69	0.48	0.39	0.41
Campaigns,VaccCov	0.57	0.70	0.88	0.72	0.77	-0.51	-0.07	0.72	0.73	0.85	0.47
Dog density,Dog presence	0.86	**0.91**	0.73	0.82	0.75	-0.17	0.06	0.68	0.66	0.63	0.09
Dog density,Elevation	0.47	0.65	0.35	0.44	0.38	0.43	0.28	0.47	-0.05	0.36	0.37
Dog density,HDR	-0.01	0.14	0.10	0.03	0.17	-0.32	-0.03	0.01	-0.08	-0.04	0.28
Dog density,Rivers	0.14	0.34	-0.22	0.41	0.39	-0.10	-0.18	0.27	0.04	0.21	-0.02
Dog density,Roads	0.22	0.69	0.69	0.26	0.37	0.59	-0.19	0.52	0.66	0.51	0.51
Dog density,Slope	0.78	0.61	0.22	0.54	0.21	-0.67	-0.47	0.05	0.35	0.21	-0.54
Dog density,Susceptibles	**0.97**	**0.99**	**0.98**	**0.98**	**0.98**	**0.96**	**0.98**	**0.96**	**0.96**	**0.97**	**0.98**
Dog density,VaccCov	0.30	0.55	0.23	0.31	0.36	-0.22	0.28	0.20	0.09	0.40	0.38
Dog presence,Elevation	0.85	0.86	0.77	0.80	0.83	0.11	0.15	**0.90**	0.00	0.86	0.14
Dog presence,HDR	0.19	0.20	-0.10	0.03	0.20	0.04	0.11	0.38	-0.17	0.03	0.51
Dog presence,Rivers	0.24	0.59	-0.13	0.75	0.78	0.57	0.51	0.73	0.00	0.08	0.55
Dog presence,Roads	0.34	0.66	0.84	0.26	0.44	-0.06	0.26	0.57	0.54	0.63	0.19
Dog presence,Slope	0.67	0.67	0.29	0.76	0.54	0.38	0.43	0.19	0.61	0.56	0.08
Dog presence,Susceptibles	0.88	**0.93**	0.74	0.82	0.73	-0.16	0.06	0.70	0.64	0.66	0.12

MOLECULAR ECOLOGY

Dog presence,VaccCov	0.45	0.53	0.51	0.06	0.11	-0.16	0.12	0.36	0.22	0.33	-0.12
Elevation,HDR	0.42	0.55	-0.18	0.39	0.49	0.32	-0.11	0.60	0.65	0.09	0.20
Elevation,Rivers	0.26	0.78	0.10	0.75	0.79	0.25	0.26	0.71	0.35	0.04	0.20
Elevation,Roads	0.52	0.52	0.46	0.49	0.57	0.41	-0.06	0.54	0.03	0.49	0.45
Elevation,Slope	0.28	0.41	0.15	0.47	0.33	0.10	0.30	0.14	0.02	0.36	-0.04
Elevation,Susceptibles	0.51	0.69	0.42	0.52	0.43	0.46	0.33	0.55	-0.08	0.41	0.43
Elevation,VaccCov	0.49	0.55	0.74	0.09	0.17	0.37	0.31	0.48	-0.15	0.39	0.52
HDR,Rivers	0.42	0.32	-0.03	0.10	0.31	0.42	0.24	0.36	0.14	0.05	0.24
HDR,Roads	0.51	0.21	0.05	0.21	0.23	-0.03	-0.21	0.28	-0.11	-0.13	-0.09
HDR,Slope	-0.49	-0.42	0.08	-0.37	-0.48	0.35	0.06	-0.33	-0.10	0.05	-0.05
HDR,Susceptibles	0.14	0.21	0.10	0.15	0.32	-0.16	-0.02	0.21	-0.12	-0.07	0.27
HDR,VaccCov	**0.91**	0.75	-0.13	0.73	0.79	0.90	0.15	0.80	-0.30	0.10	0.24
Rivers,Roads	-0.29	0.06	-0.40	0.09	0.22	-0.07	0.23	0.33	-0.08	0.16	0.26
Rivers,Slope	0.16	0.51	0.35	0.52	0.35	0.51	0.60	0.25	-0.04	-0.09	0.50
Rivers,Susceptibles	0.28	0.34	-0.28	0.42	0.39	-0.01	-0.14	0.28	-0.06	0.33	0.04
Rivers,VaccCov	0.43	0.10	-0.06	-0.13	0.02	0.24	-0.03	0.13	0.23	0.44	0.01
Roads,Slope	-0.32	0.06	0.04	-0.13	0.00	-0.57	-0.09	-0.07	-0.03	0.03	-0.19
Roads,Susceptibles	0.14	0.70	0.75	0.33	0.45	0.63	-0.11	0.63	0.72	0.60	0.58
Roads,VaccCov	0.40	0.54	0.43	0.02	0.06	0.10	-0.23	0.19	0.36	0.06	0.15
Slope,Susceptibles	0.75	0.57	0.08	0.51	0.11	-0.67	-0.51	-0.09	0.22	0.15	-0.53
Slope,VaccCov	-0.16	-0.16	-0.40	-0.13	-0.36	0.29	-0.25	-0.34	-0.41	-0.11	-0.24
Susceptibles,VaccCov	0.48	0.64	0.36	0.35	0.44	-0.08	0.32	0.37	0.24	0.49	0.45

MOLECULAR ECOLOGY

S3 Table. Epidemiological information and whole genome sequencing (WGS) details for 152 rabies virus samples collected from the Serengeti District, Tanzania, between 2004 and 2013.

Sample ID	Date of sample collection	Easting	Northing	Host	WGS protocol	Total quality trimmed reads	Viral reads mapped	Proportion viral reads	Average depth	GenBank Accession	Details/Reference
RV2483	28/09/08	701590	9794710	Goat	MiSeq	397221	2045	0.51	22.05	KY210220	This paper
RV2485	28/09/08	701590	9794710	Goat	MiSeq	300966	907	0.30	8.12	KY210221	This paper
RV2489	17/01/09	699900	9794413	Domestic dog	MiSeq	2665913	3359	0.13	16.43	KY210222	This paper
RV2490	07/08/08	702122	9796402	Domestic dog	MiSeq	1013605	8526	0.84	79.09	KR534217	Original GenBank submission (Brunker *et al* ,2015) updated to WGS for this paper
RV2491	02/08/08	696748	9794246	Domestic dog	MiSeq	1421454	10733	0.76	101.98	KR534218	Original GenBank submission (Brunker

											et al ,2015) updated to WGS for this paper
RV2492	03/11/07	685837	9799418	Domestic dog	MiSeq	1642925	12895	0.78	100.93	KR534219	Original GenBank submission (Brunker *et al* ,2015) updated to WGS for this paper
RV2493	31/07/08	700689	9793979	Domestic dog	MiSeq	1304543	4780	0.37	44.57	KR534220	Original GenBank submission (Brunker *et al* ,2015) updated to WGS for this paper
RV2495	12/07/08	696385	9802796	Domestic dog	MiSeq	1946750	14768	0.76	129.48	KR906734	Brunker *et al* ,2015

RV2497	22/06/07	678577	9807715	Domestic dog	MiSeq/NextSeq	15591341	6031	0.04	63.98	KY210223	This paper
RV2500	23/11/08	698019	9804712	Domestic dog	MiSeq	557886	1536	0.28	15.86	KR906737	Brunker *et al* ,2015
RV2501	14/02/04	679372	9810508	Domestic dog	MiSeq	1030023	11539	1.12	108.23	KR906738	Brunker *et al* ,2015
RV2503	15/02/09	686729	9761151	Wild cat	MiSeq	1325075	2774	0.21	28.64	KR906740	Brunker *et al* ,2015
RV2767	12/09/09	665178	9777064	Domestic dog	MiSeq	1039525	23560	2.27	249.79	KR906742	Brunker *et al* ,2015
RV2786	20/06/10	670876	9811501	Domestic dog	MiSeq/NextSeq	15706254	10705	0.07	109.00	KY210224	This paper
RV2788	05/08/10	668396	9794012	Domestic dog	MiSeq	1952400	16416	0.84	181.53	KR534228	Original GenBank submission (Brunker *et al* ,2015) updated to WGS for this paper
RV2789	22/10/10	686741	9795339	Domestic	MiSeq	825552	1753	0.21	16.94	KR534229	Original GenBank

				dog							submission (Brunker *et al* ,2015) updated to WGS for this paper
RV2790	01/11/10	678362	9807838	Domestic dog	MiSeq	190118	2054	1.08	20.87	KR534230	Original GenBank submission (Brunker *et al* ,2015) updated to WGS for this paper
RV2791	08/11/10	681329	9802616	Domestic dog	MiSeq/NextSeq	15068608	20740	0.14	237.54	KR534231	Original GenBank submission (Brunker *et al* ,2015) updated to WGS for this paper

RV2792	07/01/11	670624	9824613	Jackal	MiSeq/NextSeq	13995380	19366	0.14	215.26	KR534232	Original GenBank submission (Brunker *et al* ,2015) updated to WGS for this paper
RV2793	02/01/11	657176	9822908	Cow	MiSeq	149085	3900	2.62	38.81	KR906755	Brunker *et al* ,2015
RV2794	12/01/11	679391	9807221	Domestic dog	MiSeq	514797	2064	0.40	19.28	KR534233	Original GenBank submission (Brunker *et al* ,2015) updated to WGS for this paper
RV2795	16/01/11	682879	9801080	Cow	MiSeq	369231	12824	3.47	134.33	KR534234	Original GenBank submission (Brunker *et al*

											,2015) updated to WGS for this paper
RV27 96	19/01/11	671 651	9801 501	Cow	MiSeq	1033943	56028	5.42	508.13	KR53 4235	Original GenBank submissio n (Brunker *et al* ,2015) updated to WGS for this paper
RV27 97	30/01/11	663 782	9800 181	Cow	MiSeq	1278027	25740	2.01	229.21	KR53 4236	Original GenBank submissio n (Brunker *et al* ,2015) updated to WGS for this paper
RV27 98	31/01/11	663 782	9800 181	Cow	MiSeq	1287235	69613	5.41	604.45	KR53 4237	Original GenBank submissio n

											(Brunker *et al* ,2015) updated to WGS for this paper
RV2799	29/01/11	680129	9805797	Domestic dog	MiSeq	1347238	4352	0.32	41.33	KR906756	Brunker *et al* ,2015
RV2800	17/02/11	666237	9778079	Domestic dog	MiSeq	142582	2917	2.05	33.48	KR534238	Original GenBank submission (Brunker *et al* ,2015) updated to WGS for this paper
RV2858	21/02/11	646139	9824316	Goat	MiSeq	1503125	120336	8.01	935.41	KY210225	This paper
RV2859	01/03/11	650148	9819597	Domestic dog	MiSeq	283796	8437	2.97	88.54	KY210226	This paper
RV2861	11/05/11	691423	9791388	Domestic dog	MiSeq	1359627	164027	12.06	1259.37	KR906767	Brunker *et al* ,2015
RV2862	11/05/11	674259	9805687	Domestic	MiSeq	1177250	2121	0.18	21.18	KR906768	Brunker *et al*

				dog							,2015
RV28 63	19/04/11	672 066	9805 512	Dom estic dog	MiSeq	448320	24262	5.41	194.02	KY21 0227	This paper
RV28 66	15/04/11	678 461	9808 213	Goat	MiSeq/NextSeq	17707815	1007	0.01	9.98	KY21 0228	This paper
RV28 67	22/05/11	677 206	9802 038	Dom estic dog	454 pyrosequencing: depletion	2965005	2967	0.10	18.65	KY21 0229	This paper
RV28 68	06/06/11	678 588	9807 873	Cow	MiSeq	703095	27276	3.88	272.91	KY21 0230	This paper
RV28 70	01/06/11	697 216	9792 726	Dom estic dog	MiSeq	529286	19381	3.66	154.52	KY21 0231	This paper
RV28 71	17/06/11	701 315	9794 641	Dom estic dog	454 pyrosequencing: amplicon	46796	8850	18.91	25.34	KR90 6769	Brunker *et al* ,2015
RV28 73	20/06/11	687 042	9796 792	Dom estic dog	MiSeq	2235772	35378	1.58	359.16	KY21 0232	This paper
RV28 75	29/06/11	680 196	9811 835	Dom estic dog	MiSeq	407187	9927	2.44	99.27	KR90 6770	
RV28 77	30/06/11	679 837	9808 655	Goat	MiSeq	4169008	168447	4.04	1494.91	KY21 0233	This paper
RV28 78	03/07/11	689 579	9796 947	Dom estic dog	MiSeq	146105	1933	1.32	21.85	KY21 0234	This paper
RV28 79	10/06/11	698 379	9806 085	Dom estic dog	MiSeq	696671	6806	0.98	68.17	KY21 0235	This paper

MOLECULAR ECOLOGY

RV2880	02/07/11	678588	9807873	Cow	MiSeq	795216	8731	1.10	92.11	KY210236	This paper
RV2881	07/07/11	698357	9806963	Cow	MiSeq	1101481	12573	1.14	117.68	KY210237	This paper
RV2882	23/07/11	676741	9807751	Domestic dog	454 pyrosequencing: depletion	2896363	35151	1.21	88.58	KY210238	This paper
RV2883	23/07/11	686525	9797308	Domestic dog	MiSeq/NextSeq	20693938	21557	0.10	221.41	KY210239	This paper
RV2884	21/07/11	675366	9806739	Cow	MiSeq	1107607	9926	0.90	102.65	KY210240	This paper
RV2885	24/07/11	694855	9798730	Goat	MiSeq	969814	3303	0.34	33.06	KY210241	This paper
RV2886	21/07/11	701234	9794942	Goat	454 pyrosequencing: amplicon	48056	16226	33.76	64.02	KY210242	This paper
RV2887	25/07/11	695864	9798032	Domestic dog	MiSeq	1044324	2444	0.23	24.46	KY210243	This paper
RV2888	29/07/11	700262	9793490	Domestic dog	MiSeq	1517717	19413	1.28	189.29	KY210244	This paper
RV2889	03/08/11	693407	9791698	Domestic dog	MiSeq	1395952	5503	0.39	50.33	KR534244	Original GenBank submission (Brunker *et al* ,2015) updated

											to WGS for this paper
RV28 90	03/08/11	651 666	9819 798	Cow	MiSeq	1725539	16406	0.95	155.06	KR53 4245	Original GenBank submissio n (Brunker *et al* ,2015) updated to WGS for this paper
RV28 91	10/08/11	680 536	9813 026	Civet	MiSeq	1290933	54121	4.19	414.53	KR53 4246	Original GenBank submissio n (Brunker *et al* ,2015) updated to WGS for this paper
RV28 92	12/08/11	676 741	9807 751	Dom estic dog	454 pyrosequencing: depletion	2796394	50188	1.79	91.09	KR53 4247	Original GenBank submissio n (Brunker *et al*

											,2015) updated to WGS for this paper
RV28 93	01/08/11	651 666	9819 798	Cow	MiSeq	1637158	31071	1.90	278.08	KR53 4248	Original GenBank submissio n (Brunker *et al* ,2015) updated to WGS for this paper
RV28 94	15/08/11	653 024	9822 353	Dom estic dog	MiSeq	2125096	188049	8.85	1469.55	KR90 6771	Brunker *et al* ,2015
RV28 95	18/08/11	678 334	9797 356	Dom estic dog	MiSeq	2273420	19720	0.87	210.02	KR53 4249	Original GenBank submissio n (Brunker *et al* ,2015) updated to WGS for this paper
RV28	19/08/11	681	9798	Dom	MiSeq	1104977	7214	0.65	68.67	KR90	Brunker

96		869	035	estic dog						6772	*et al* ,2015
RV2897	02/09/11	678907	9808303	Domestic dog	MiSeq	984501	9581	0.97	92.68	KR534250	Original GenBank submission (Brunker *et al* ,2015) updated to WGS for this paper
RV2898	18/09/11	658269	9804143	Domestic dog	MiSeq/NextSeq	19197193	20664	0.11	206.05	KR534251	Original GenBank submission (Brunker *et al* ,2015) updated to WGS for this paper
RV2899	23/09/11	658245	9804462	Domestic dog	MiSeq	897751	8818	0.98	85.53	KR534252	Original GenBank submission (Brunker *et al* ,2015)

											updated to WGS for this paper
RV2900	27/09/11	656396	9803751	Domestic dog	MiSeq	1326869	8924	0.67	84.32	KR906773	Brunker *et al* ,2015
RV2901	22/09/11	653179	9802910	Domestic dog	MiSeq	522728	10710	2.05	106.87	KR906774	Brunker *et al* ,2015
RV2902	24/09/11	684532	9790009	Domestic dog	MiSeq	1204863	5234	0.43	43.68	KR906775	Brunker *et al* ,2015
RV2903	05/10/11	657232	9804521	Domestic dog	MiSeq	1219764	6605	0.54	62.45	KR534253	Original GenBank submission (Brunker *et al* ,2015) updated to WGS for this paper
RV2906	08/09/11	681338	9808012	Cat	MiSeq	1283722	19254	1.50	170.85	KR534254	Original GenBank submission (Brunker *et al* ,2015)

MOLECULAR ECOLOGY

											updated to WGS for this paper
RV2907	16/10/11	700560	9803728	Cow	MiSeq	1353429	7867	0.58	71.47	KR906776	Brunker *et al* ,2015
RV2909	12/11/11	663440	9804477	Cow	MiSeq	893349	20317	2.27	194.08	KR534256	Brunker *et al* ,2015
RV3047	10/08/12	655560	9809040	Domestic dog	NextSeq	14822146	193963	1.31	1634.14	KY210245	This paper
RV3048	17/09/12	690718	9803251	Sheep	NextSeq	13774437	92882	0.67	865.92	KY210246	This paper
RV3049	04/10/12	689087	9797146	Domestic dog	NextSeq	14925758	68578	0.46	634.13	KY210247	This paper
RV3050	02/09/12	671593	9804068	Domestic dog	NextSeq	12716032	689856	5.43	5022.73	KY210248	This paper
RV3051	12/10/12	684769	9779316	Domestic dog	NextSeq	13274035	33398	0.25	308.65	KY210249	This paper
RV3052	13/11/12	643810	9813739	Domestic dog	NextSeq	10917330	110185	1.01	1077.20	KY210250	This paper
RV3053	10/11/12	646262	9815621	Domestic dog	NextSeq	11787802	61379	0.52	600.13	KY210251	This paper
RV30	08/12/12	655	9810	Dom	NextSeq	13502719	629341	4.66	4329.86	KY21	This paper

57		999	468	estic dog						0252	
RV30 58	18/01/13	648 713	9819 325	Dom estic dog	NextSeq	11873546	143723	1.21	1031.42	KY21 0253	This paper
RV30 59	11/01/13	654 132	9825 734	Dom estic dog	NextSeq	12420277	786476	6.33	4587.68	KY21 0254	This paper
RV30 60	04/01/13	654 509	9823 226	Dom estic dog	NextSeq	12150398	316716	2.61	2450.02	KY21 0255	This paper
RV30 61	08/01/13	651 604	9821 249	Dom estic dog	NextSeq	12380235	386682	3.12	2883.85	KY21 0256	This paper
RV30 63	08/01/13	691 639	9810 370	Dom estic dog	NextSeq	11500684	37364	0.32	365.20	KY21 0257	This paper
RV30 64	02/01/13	687 567	9796 857	Dom estic dog	NextSeq	11829231	48612	0.41	469.26	KY21 0258	This paper
RV30 66	23/02/13	652 145	9807 506	Cat	NextSeq	10441152	159856	1.53	1487.65	KY21 0259	This paper
RV30 67	25/02/13	653 061	9777 091	Dom estic dog	NextSeq	15872978	3697	0.02	35.46	KY21 0260	This paper
RV30 68	11/04/13	696 403	9791 890	Goat	NextSeq	11386195	304247	2.67	2459.48	KY21 0261	This paper
RV30 70	27/02/13	673 866	9807 701	Goat	NextSeq	10573869	50029	0.47	504.04	KY21 0262	This paper
RV30 71	13/04/13	680 151	9814 868	Dom estic	NextSeq	13361066	1493699	11.18	7981.02	KY21 0263	This paper

MOLECULAR ECOLOGY

				dog							
RV3072	10/04/13	672356	9817934	Domestic dog	NextSeq	10909432	1088443	9.98	6940.73	KY210264	This paper
RV3073	18/02/13	697846	9808299	Domestic dog	NextSeq	9431525	84544	0.90	816.23	KY210265	This paper
RV3074	01/02/13	658498	9825235	Domestic dog	NextSeq	13487972	780515	5.79	5369.35	KY210266	This paper
RV3075	31/01/13	658498	9825235	Domestic dog	NextSeq	12309563	85376	0.69	834.07	KY210267	This paper
RV3078	26/05/13	684594	9779133	Cow	NextSeq	13267270	324339	2.44	2550.76	KY210268	This paper
RV3079	18/07/13	700214	9793780	Domestic dog	NextSeq	13140710	10369	0.08	103.78	KY210269	This paper
RV3080	17/07/13	700300	9793350	Domestic dog	NextSeq	12398079	71735	0.58	581.75	KY210270	This paper
RV3082	03/09/13	670878	9795201	Domestic dog	NextSeq	13023573	46367	0.36	485.26	KY210271	This paper
RV3084	22/08/13	697865	9803607	Domestic dog	NextSeq	9206525	18253	0.20	179.18	KY210272	This paper
RV3085	06/09/13	683848	9810611	Domestic dog	NextSeq	11789633	64210	0.54	590.80	KY210273	This paper

MOLECULAR ECOLOGY

RV3086	06/09/13	697902	9792902	Cow	NextSeq	13831121	298860	2.16	2439.13	KY210274	This paper
RV3087	06/09/13	699819	9795099	Cow	NextSeq	17902151	12527	0.07	126.18	KY210275	This paper
RV3088	25/04/13	691229	9812934	Cow	NextSeq	18221434	358845	1.97	2936.54	KY210276	This paper
RV3090	25/11/11	696825	9790124	Cow	MiSeq	129980	10825	8.33	104.41	KY210277	This paper
RV3091	05/12/11	696698	9792023	Domestic dog	MiSeq/NextSeq	18376860	50860	0.28	496.86	KR906777	Brunker *et al* ,2015
RV3092	16/12/11	680305	9798935	Domestic dog	MiSeq	1045220	2104	0.20	18.69	KY210278	This paper
RV3093	22/12/11	681720	9798404	Domestic dog	MiSeq	495576	4371	0.88	42.70	KR906778	Brunker *et al* ,2015
RV3094	21/12/11	699127	9791922	Domestic dog	MiSeq	1100604	3942	0.36	35.79	KY210279	This paper
RV3096	30/01/12	658200	9804251	Domestic dog	MiSeq	758254	2900	0.38	24.44	KY210280	This paper
RV3097	19/01/12	658323	9804533	Domestic dog	MiSeq	384498	12069	3.14	113.75	KY210281	This paper
RV3098	26/01/12	658761	9804127	Domestic dog	MiSeq	277003	7448	2.69	73.18	KY210282	This paper
RV3099	19/01/12	696747	9790182	Cow	MiSeq	1440888	30135	2.09	233.29	KY210283	This paper

RV3100	19/02/12	698140	9804256	Domestic dog	MiSeq	668576	2648	0.40	24.11	KR906779	Brunker *et al* ,2015
RV3101	06/03/12	695255	9803449	Jackal	MiSeq	416501	10348	2.48	86.78	KY210284	Brunker *et al* ,2015
RV3102	08/03/12	700597	9803335	Goat	MiSeq	291477	13309	4.57	123.32	KY210285	Brunker *et al* ,2015
RV3103	10/12/11	653049	9821771	Domestic dog	MiSeq/NextSeq	17549986	11210	0.06	109.93	KY210286	Brunker *et al* ,2015
RV3104	22/12/11	648913	9823033	sheep	MiSeq	1145840	11000	0.96	95.11	KR906780	Brunker *et al* ,2015
RV3105	22/03/12	661920	9803801	Domestic dog	MiSeq	1009153	1365	0.14	13.11	KY210287	This paper
RV3107	09/04/12	656704	9803322	Domestic dog	MiSeq	959083	31920	3.33	292.64	KR906781	Brunker *et al* ,2015
RV3109	12/04/12	656691	9803564	Domestic dog	MiSeq	409667	24684	6.03	259.04	KY210288	This paper
RV3110	30/04/12	686063	9798923	Domestic dog	MiSeq	346269	6180	1.78	65.54	KY210289	This paper
RV3111	29/04/12	650984	9807685	Domestic dog	MiSeq	897329	2674	0.30	26.85	KR906782	
RV31	06/05/12	685	9792	Dom	MiSeq/NextSeq	18115513	68797	0.38	761.18	KY21	This paper

13		149	574	estic dog						0290	
RV31 14	06/05/12	685 205	9792 549	Jacka l	MiSeq	854689	16344	1.91	159.71	KY21 0291	This paper
RV31 15	11/05/12	680 871	9782 055	Dom estic dog	MiSeq	800250	3034	0.38	29.22	KY21 0292	This paper
RV31 17	12/05/12	669 765	9795 977	Dom estic dog	MiSeq	2331749	6102	0.26	57.60	KR90 6783	Brunker *et al* ,2015
RV31 19	17/05/12	680 871	9782 055	Dom estic dog	MiSeq/NextSeq	15519701	7634	0.05	81.87	KY21 0293	This paper
RV31 21	28/05/12	655 915	9809 872	Dom estic dog	MiSeq	1321266	14930	1.13	132.03	KY21 0294	This paper
RV31 22	14/05/12	662 827	9818 718	Dom estic dog	MiSeq	1328914	5972	0.45	56.12	KY21 0295	This paper
RV31 23	26/04/12	647 442	9799 177	Dom estic dog	MiSeq	1530487	7221	0.47	65.08	KR90 6792	Brunker *et al* ,2015
RV31 24	07/05/12	657 028	9804 565	Dom estic dog	MiSeq	903958	9073	1.00	86.74	KY21 0296	This paper
RV31 25	06/06/12	658 003	9809 433	Goat	MiSeq/NextSeq	16181484	10504	0.06	105.89	KR90 6784	Brunker *et al* ,2015
RV31 27	11/06/12	681 576	9797 817	Dom estic dog	MiSeq	461052	20729	4.50	204.27	KR90 6785	Brunker *et al* ,2015

MOLECULAR ECOLOGY

RV3128	16/06/12	674458	9797076	Donkey	MiSeq	2044844	9514	0.47	88.78	KR906786	Brunker *et al* ,2015
RV3129	25/06/12	662827	9819718	Sheep	MiSeq	871812	41446	4.75	408.32	KY210297	This paper
RV3130	28/06/12	662827	9818718	Goat	MiSeq	155506	10100	6.49	103.35	KY210298	This paper
RV3131	07/07/12	685498	9797037	Domestic dog	MiSeq	1518274	12181	0.80	113.24	KR906787	Brunker *et al* ,2015
RV3132	02/04/12	656028	9801566	Domestic dog	MiSeq	489623	1478	0.30	13.93	KR906788	Brunker *et al* ,2015
RV3133	04/07/12	700856	9800221	Domestic dog	MiSeq/NextSeq	14589139	11715	0.08	118.01	KR906789	Brunker *et al* ,2015
RV3134	19/07/12	689067	9791597	Domestic dog	MiSeq	1280905	3509	0.27	34.25	KY210299	This paper
RV3135	19/07/12	695591	9788282	Domestic dog	MiSeq	1327972	10241	0.77	95.70	KY210300	This paper
RV3136	16/07/12	678119	9799572	Cow	MiSeq	993550	15864	1.60	153.90	KY210301	This paper
RV3137	21/07/12	682623	9783025	Goat	MiSeq	673235	14221	2.11	140.50	KY210302	This paper
RV3138	16/07/12	677206	9805047	Domestic dog	MiSeq	1634873	13081	0.80	126.10	KY210303	This paper
RV3139	07/07/12	685498	9797937	Domestic	MiSeq	1500822	7764	0.52	74.40	KY210304	This paper

				dog							
RV3140	27/07/12	656945	9804374	Domestic dog	MiSeq	1907863	99500	5.22	737.59	KR906790	Brunker *et al* ,2015
RV3145	08/08/12	658701	9825071	Hyena	MiSeq/NextSeq	15216797	10232	0.07	110.25	KY210305	This paper
RV3146	26/05/12	697865	9803637	Civet	MiSeq/NextSeq	16819682	13702	0.08	135.45	KY210306	This paper
RV3149	23/01/12	657742	9809576	Domestic dog	MiSeq	365335	802	0.22	6.23	KR906791	This paper
RV3150	24/03/12	682197	9791269	Domestic dog	MiSeq	1194163	43373	3.63	407.75	KY210307	This paper
RV3151	07/01/12	648913	9823033	Cow	MiSeq	105666	6227	5.89	60.67	KY210308	This paper
RV3152	31/05/12	695002	9803207	NA	MiSeq/NextSeq	14910632	4003	0.03	42.77	KY210309	This paper
RV3153	23/01/12	694625	9806377	Goat	NextSeq	12054558	62937	0.52	637.73	KY210310	This paper
RV3154	29/04/12	658670	9802617	Domestic dog	NextSeq	14618752	401835	2.75	3228.77	KY210311	This paper

MOLECULAR ECOLOGY

S1 Fig. Pearson's correlation between human and dog population density in the Serengeti District based on a complete census conducted in the district.

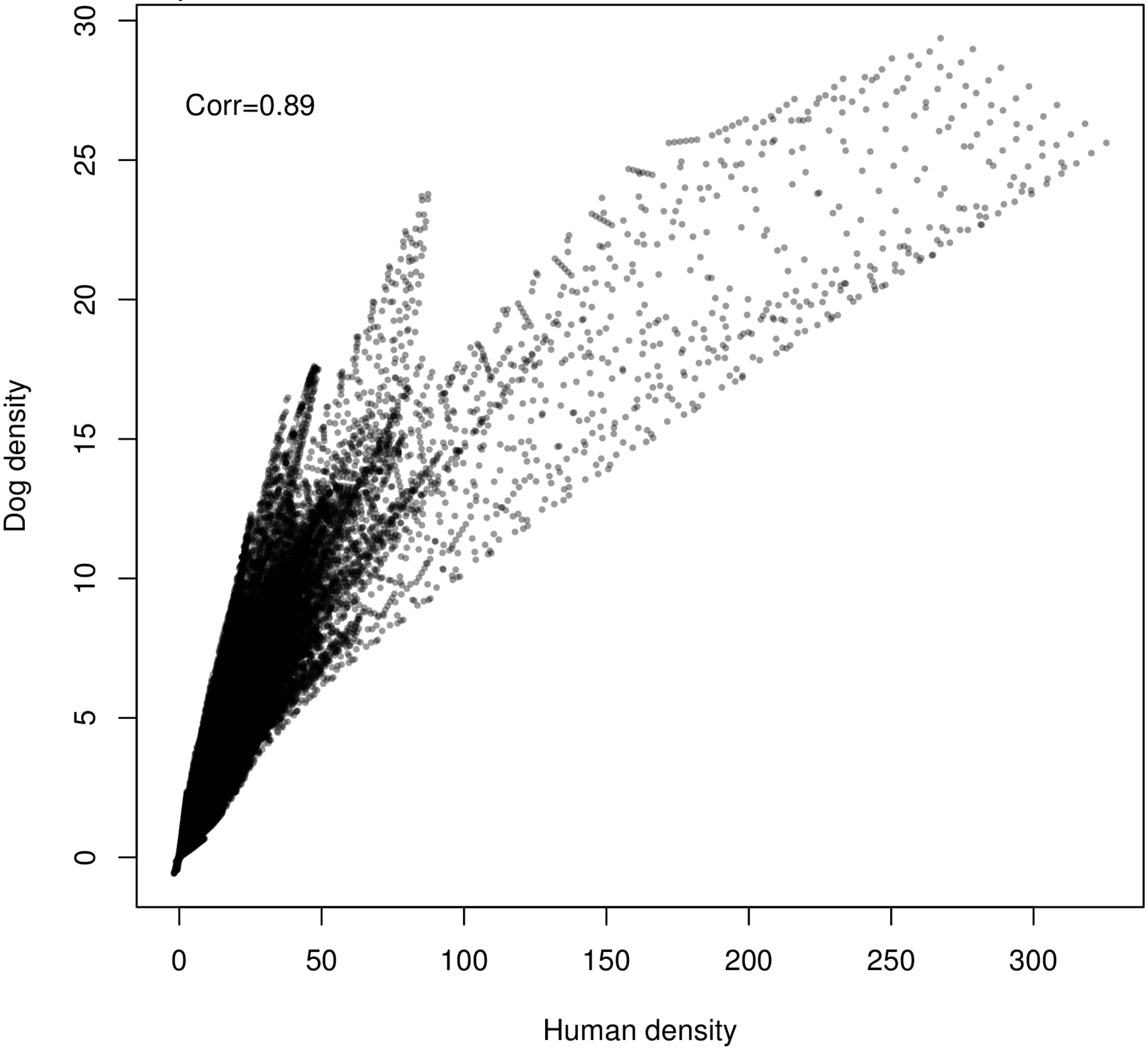

MOLECULAR ECOLOGY

S2 Fig. Maximum likelihood phylogeny of 152 rabies cases collected from the Serengeti District, Tanzania 2004-2013. The phylogeny is scaled in substitutions per site and two major lineages are coloured, Tz1 (yellow triangles) and Tz3 (purple circles). Grey circles indicate bootstrap support ≥80%.

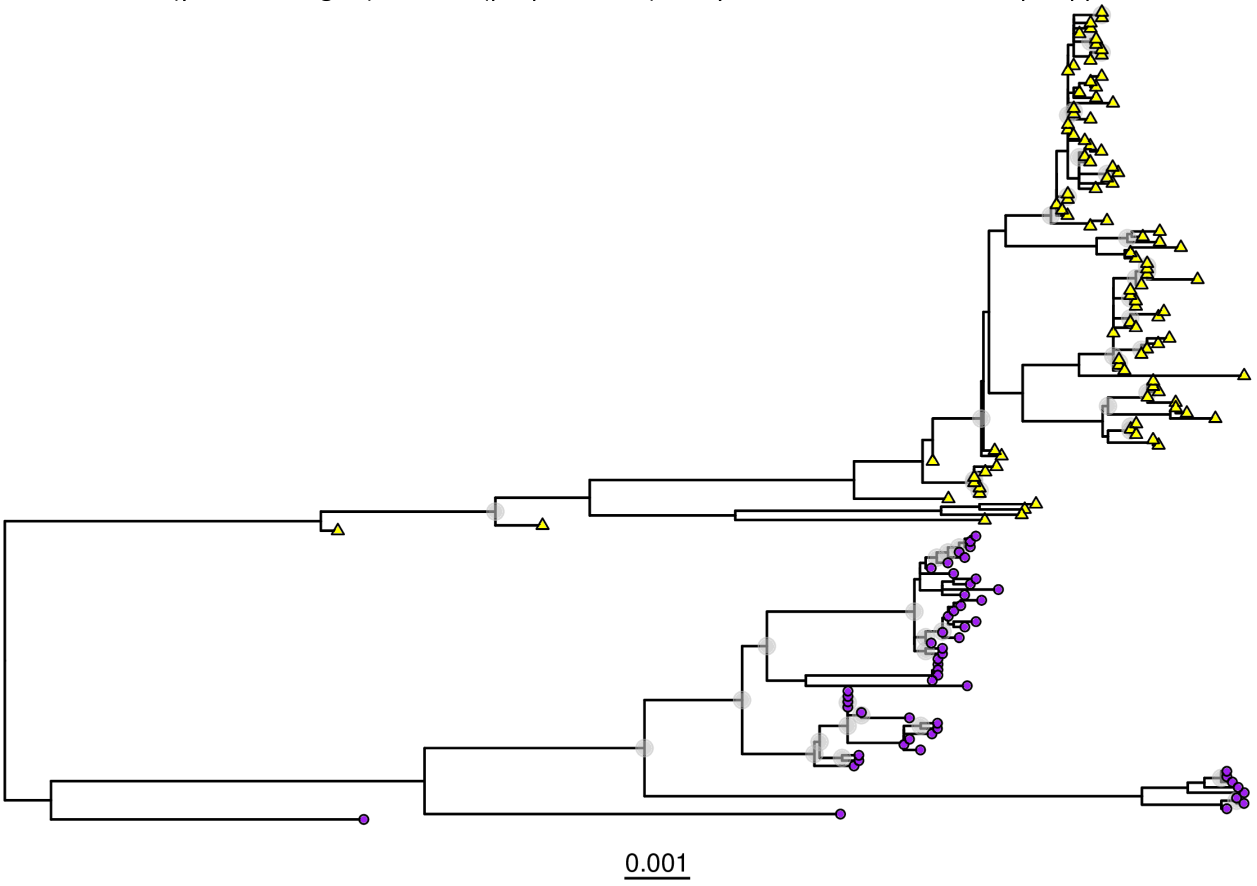

MOLECULAR ECOLOGY

S3 Fig. Spatial clusters of 152 rabies cases in the Serengeti District according to k-means partitioning using a Euclidean distance matrix. (A-K) Each cluster is designated by a unique colour/symbol combination for a number of clusters (*k*) ranging from *k*=5 to *k*=15. Map created using R packages ggmap (Kahle & Wickham, 2013) and maptools (Lewin-Koh et al., 2012) with administrative boundaries from www.nbs.go.tz.

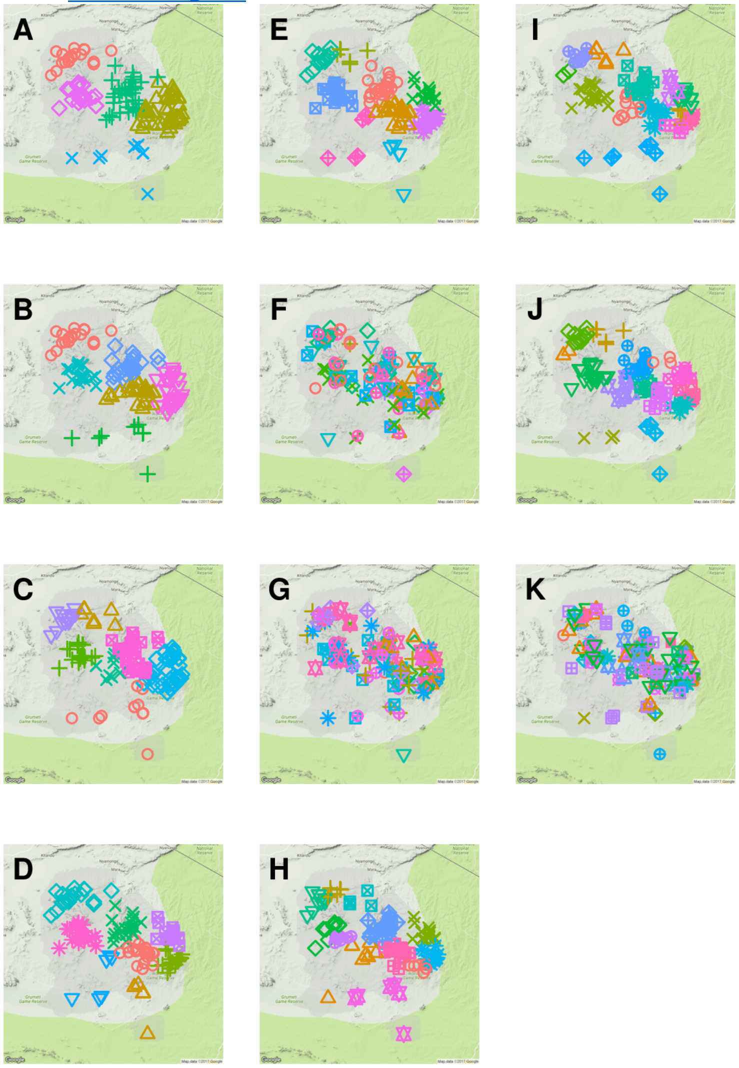

MOLECULAR ECOLOGY

S4 Fig. Centroids of spatial clusters assigned according to according to k-means partitioning using a Euclidean distance matrix for 152 rabies cases in the Serengeti District. (A-K) Centroid positions for a number of clusters (*k*) ranging from *k*=5 to *k*=15. Map created using R package maptools and administrative boundaries from www.nbs.go.tz.

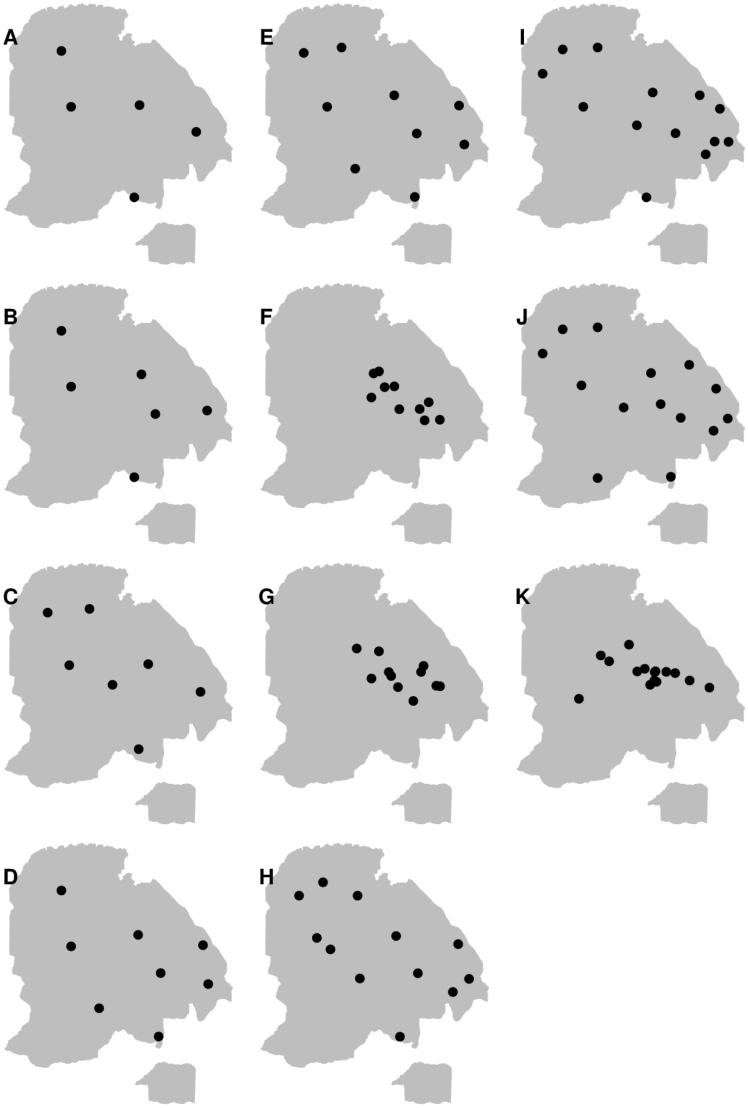

Supplement: Supplementary file 1 [file MEC-27-773-s001.pdf]
